# Discovery of a mammalian FASN inhibitor against xenografts of non-small cell lung cancer and melanoma

**DOI:** 10.1038/s41392-022-01099-4

**Published:** 2022-08-24

**Authors:** Danfeng Cao, Jie Yang, Youchao Deng, Meng Su, Yeji Wang, Xueqiong Feng, Yi Xiong, Enhe Bai, Yanwen Duan, Yong Huang

**Affiliations:** 1 Xiangya International Academy of Translational Medicine at Central South University, Changsha, Hunan 410013 China; 2 Xiangya Hospital at Central South University, Changsha, Hunan 410013 China; 3 National Engineering Research Center of Combinatorial Biosynthesis for Drug Discovery, Changsha, Hunan 410011 China

**Keywords:** Translational research, Drug screening

**Dear Editor**,

Cancer cells need to reprogram fatty acid (FA) metabolism to promote cell growth and survival through exogenous lipid uptake and FA biosynthesis catalyzed by the multidomain containing mammalian FA synthase (FASN).^1,2^ The bidirectional relationships of oncogenic signaling and *de novo* lipogenesis (DNL) suggest that FASN is a druggable target in many cancers. Although FASN inhibitors including Fasnall, GSK2194069, IPI-9119, orlistat, TVB-2640, TVB-3166, and TVB-3664 have shown promise in preclinical cancer models or early-phase clinical trials, none have been approved for the treatment of cancers (Supplementary Table S1).^3^ Here we report a unique inhibitor targeting the ketosynthase (KS) domain of FASN, which shows superior cytotoxicity and selectivity over orlistat and TVB-3166, as well as strong antitumor effects in both non-small cell lung cancer (NSCLC) and melanoma mouse xenografts.

Platensimycin was originally discovered as a bacterial FabF/B inhibitor by competition with their substrate malonyl-acyl carrier protein (ACP), while later it showed potent inhibitory activity (IC_50_ = 0.3 μM) against mammalian FASN.^4^ To discover selective FASN inhibitors against NSCLC, we screened a focused library of platensimycin derivatives against KRAS-positive A549 and NCI-H1299 cells (Fig. 1a and Supplementary Figs. S1–S6). Several 6-aryl platensimycin derivatives showed inhibitory activity from 54.3% to 91.2% against both cell lines (Supplementary Fig. S7), among which **6p** exhibited an IC_50_ of 16.95 ± 1.96 μM, superior to TVB-3166, orlistat, and cerulenin (Fig. 1b and Supplementary Table S2). The selectivity of **6p** may protect the less fatty acid-dependent normal cells upon its exposure. The **6p**-treated cells showed notable morphological changes, reduced colony number and sizes either alone or in combination with cis-platinum (CDDP) (Fig. 1c and Supplementary Fig. S8a–b), as well as G2/M cell cycle arrest and apoptosis (Supplementary Fig. S8c–e). Furthermore, **6p** appreciably inhibited cell migration and invasion (Fig. 1d and Supplementary Fig. S9). In contrast, 6-(4-bromophenyl)-platensic acid and platensimycin showed no cytotoxicity, suggesting that 3-amino-2,4-dihydroxybenzoic acid (ADHBA) and 6-(4-bromophenyl) group are critical for the cytotoxicity of **6p** (Supplementary Fig. S10).

**Fig. 1:**
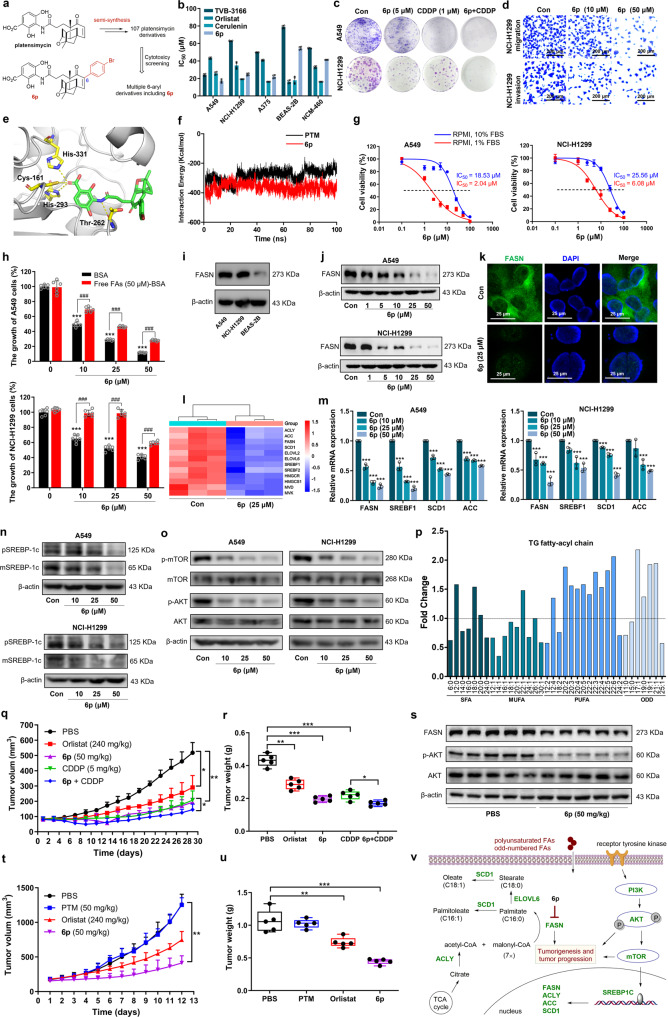
Discovery of **6p** against NSCLC and melanoma. **a** Identification of **6p** from 107 semisynthetic platensimycin derivatives prepared in our previous studies. **b** IC_50_ values of FASN inhibitors TVB-3166, orlistat, cerulenin, and **6p** against cancer cell lines A549, NCI-H1299, A375, as well as normal cell lines BEAS-2B and NCM-460. The selectivities of **6p** against A549 and NCI-H1299 towards normal cells were 3.21 and 2.21, respectively (*n* = 4). **c** Inhibition of colony formation in A549 and NCI-H1299 cells treated with **6p** alone or in combination with cis-platinum (CDDP) (*n* = 3). **d** Inhibition of NCI-H1299 cell migration and invasion by **6p** in the transwell assay (*n* = 3). **e** The predicted docking mode of **6p** (green) in human FASN KS-MAT didomain (PDB ID: 3HHD). **f** Molecular dynamics simulation showing the non-bond interaction energies. **6p** was in red and platensimycin (PTM) was in black. **g** The IC_50_ values of **6p** against A549 and NCI-H1299 cells in RPMI medium containing 10% (blue) or 1% fetal bovine serum (red). **h** Cell growth was rescued by exogenous free FAs (oleic acid: palmitic acid = 2:1), Two-way ANOVA test (*n* = 6), ^###^*p* < 0.001 **6p** + free FAs versus **6p**. **i** Determinat**i**on of FASN expression levels in A549, NCI-H1299, and BEAS-2B. **j** Western blotting showed the decreased FASN expression by **6p** treatment in A549 and NCI-H1299 cell lines (n = 3). **k** The reduced FASN expression in cytoplasm under confocal microscopy in NCI-H1299 cells treated by **6p**. **l** RNA-seq analysis of NCI-H1299 cells treated with **6p** (25 μM) showed down-regulation of key genes involved in DNL and cholesterol synthesis (*n* = 3). **m** RT-qPCR results showed that **6p** decreased the expression of key DNL genes, One-way ANOVA test (*n* = 3). **n** Western blotting showed the decreased SREBP-1c expression by **6p** treatment in A549 and NCI-H1299 cells. pSREBP-1c: SREBP-1c precursor; mSREBP-1c: mature forms of SREBP-1c. **o** The down-regulation of PI3K-AKT-mTOR signaling pathway by **6p** treatment in A549 and NCI-H1299 cells (*n* = 3). **p** Lipidomic analysis showed that **6p** (25 μM) changed the total intensity fold changes of individual fatty-acyl chains associated with total triacylglycerides sorted by degree of saturation. SAF, saturated fatty acyls; MUFA, monounsaturated fatty acyls; PUFA, polyunsaturated fatty acyls; ODD, odd-numbered fatty acyls (*n* = 5). **q, r** The tumor volume (**q**) and tumor weight (**r**) were significantly reduced when A549 tumor xenografts were treated with **6p**, and the combination treatment of **6p** and CDDP inhibited tumor growth synergistically. Two-way ANOVA test (**q**), One-way ANOVA test (**r**) (*n* = 5). **s** Western blotting showed that **6p** decreased the expression of p-AKT and FASN in A549 tumor xenografts (*n* = 5). **t, u** The tumor volume (**t**) and tumor weight (**u**) were significantly reduced when A375 tumor xenografts were treated with **6p**. Two-way ANOVA test (**t**), One-way ANOVA test (**u**) (*n* = 5). **v** The proposed mode of inhibition of FASN by **6p**, which decreased the expression of key lipogenic genes (labelled in green), and downregulated the PI3K-AKT-mTOR oncogenic signaling; exogenous polyunsaturated and odd-numbered FAs were transported to the cells as a compensating mechanism to sustain cell survival. Data were shown as means ± SD, *p < 0.05; **p < 0.01; ***p < 0.001

To study if **6p** interacts with FASN, it was docked to the KS-malonyltransferase (MAT) didomain of FASN, showing hydrogen bonding interactions of ADHBA with H_293_ and H_331_ of KS active site (Fig. 1e and Supplementary Fig. S10c). The molecular dynamic simulation revealed that **6p** likely bound to KS-MAT more tightly than platensimycin, with 26.76 kcal/mol less non-bond energy (Fig. 1f and Supplementary Fig. S10). The IC_50_ values of **6p** were reduced by 9- or 4-fold in A549 and NCI-H1299 cells cultured with 1% fetal bovine serum to decrease free FAs uptake, confirming the target specificity of **6p** against FASN (Fig. 1g and Supplementary Fig. S11b). Surprisingly, the cell growth inhibition could only be partially rescued by the addition of oleate and palmitate, while exogenous palmitate could rescue cell inhibition by orlistat (Fig. 1h and Supplementary Fig. S11).

FASN is highly expressed in A549 and NCI-H1299 cells, and **6p** markedly decreased its expression in a dose-dependent manner (Figs. 1i, j and Supplementary Fig. S12). Orlistat treatment only modestly affected FASN expression, while platensimycin had no obvious effects (Supplementary Fig. S13). Immunofluorescence confirmed that **6p** crucially decreased the expression of FASN in the cytoplasm of treated NCI-H1299 cells (Fig. 1k). In contrast, Fasnall or TVB-3166/IPI-9119 treatment led to either no change of FASN expression or significantly induced FASN expression, suggesting that **6p** may bypass the FASN-overexpression compensatory mechanism.^3^ RNA sequencing of **6p**-treated NCI-H1299 cells showed that **6p** significantly downregulated the expression of key genes involved in lipogenesis, cell cycle, DAN replication, mitosis, and steroid biosynthesis, and upregulated genes in apoptosis, ferroptosis, and amino acid metabolism pathways, in comparison with the control group (Fig. 1l and Supplementary Figs. S14–S15). Subsequent RT-qPCR confirmed that **6p** treatment resulted in pronounced downregulation of FASN, sterol regulatory element-binding transcription factor 1 (SREBF1), stearoyl-CoA desaturase-1 (SCD1), and acetyl-CoA carboxylase (ACC) in both A549 and NCI-H1299 cells (Fig. 1m). Immunoblotting experiments showed that **6p** dose-dependently decreased the expression of pSREBP-1c and mSREBP-1c in A549 and NCI-H1299 cells (Fig. 1n). In contrast, previous reports suggest that TVB-3166-treated tumor cells increased the expression of most of these genes.^3^ Since SREBP-1, SCD1, and ACC are all key nodes in DNL, their global down-regulation by **6p** highlights its unique antiproliferative potential.

Although the oncogenic signal transduction pathways including PI3K-AKT-mTOR regulate fatty acid biosynthesis, and oncogene KRAS activates FASN, **6p** treatment resulted in marked reduction of p-AKT and p-mTOR in the treated A549 and NCI-H1299 cells, since FA metabolism also regulates oncogenic signaling (Fig. 1o and Supplementary Fig. S16).^5^ The **6p**- or TVB-3166-treated NCI-H1299 cells had distinct lipid profiles in the HR-ESI-MS/MS analysis, exemplified by their different lipid signature in the top ten lipid metabolites, while their overall lipid composition and distribution was similar (Supplementary Figs. S17–S19 and Supplementary Table S4). The decreased total abundance of saturated and monounsaturated fatty acids include C16:0 and C18:1 in various glycerolipids and glycerophospholipids, especially triglycerides as the main forms for fatty acid storage and transport, consistent with the inhibition of FASN by **6p** (Fig. 1p, Supplementary Figs. S20–S22 and Supplementary Tables S5–S7). The significant accumulation of stearic acid (C18:0) may be caused by the down-regulation of *SCD1* (Fig. 1m). In addition, polyunsaturated fatty acid and odd-numbered fatty-acyl chains were dramatically increased because of their exogenous uptake, highlighting the metabolic flexibility of these cancer cells.

We further explored the antitumor effect of **6p** using the A549 xenograft mouse model. Subcutaneous injection of **6p** or in combination with CDDP resulted in slower tumor growth and lower tumor weight than the control group (Fig. 1q, r and Supplementary Fig. S23). HE and Ki67 staining demonstrated that **6p** effectively inhibited tumor growth with no obvious toxicity (Supplementary Fig. S23). Immuno-staining and immuno-blotting assay showed that **6p** also decreased FASN and p-AKT levels in the treated tumors (Fig. 1s and Supplementary Fig. S23e). Although FASN is also highly expressed in melanoma, few FASN inhibitors except orlistat have been evaluated in a mouse melanoma model. Since **6p** significantly inhibited clone formation and decreased FASN expression in A375 cell lines (Supplementary Fig. S24), we next evaluated its antitumor effects in A375 xenografts. **6p** effectively inhibited melanoma growth without obvious toxicity, compared to orlistat and platensimycin (Fig. 1t, u). Although intravenous administration of **6p** and platensimycin exhibited no systematic toxicity, they did not reduce tumor volume in A375-derived xenografts, which suggests the need to further optimize **6p** scaffold (Supplementary Fig. S25 and Supplementary Table S8).

In conclusion, a specific FASN inhibitor **6p** was discovered to have strong antitumor effects in two tumor xenografts through rewiring FA metabolism (Fig. 1v).^1,2^ Compound **6p** may compete with malony-ACP to inhibit KS-catalyzed Claisen condensation and block the remaining catalytic steps of other domains in FASN. The reduced production of palmitate may affect membrane architecture including lipid rafts^3^ and thus compromise activities of receptor tyrosine kinases,^5^ leading to the inhibition of PI3K-AKT-mTOR signaling and subsequent downregulation of SREBP-1, SCD1, and ACC. These lipogenic enzymes are all therapeutically explored to treat cancer and their inhibition can be achieved by **6p** treatment alone. This study not only revealed a KS-domain specific FASN inhibitor with great translational potential, but also discovered that targeting a single enzyme FASN could lead to the global inhibition of dysregulated DNL, a nearly-universal feature of cancer metabolism.

## Supplementary information

Supplemental Material

## Data Availability

The online version of this article contains supplementary material, which is available to authorized users.
