# Supplementary material for: Discovery of a mammalian FASN inhibitor against xenografts of non-small cell lung cancer and melanoma
Source: Signal Transduct Target Ther. 2022 Aug 24;7:273. doi: 10.1038/s41392-022-01099-4 (PMC9402528; doi:10.1038/s41392-022-01099-4)
Supplement: Supplementary file 1 — Supplemental Material [file 41392_2022_1099_MOESM1_ESM.docx]

**Supplemental Materials for**

**Discovery of a mammalian FASN inhibitor against xenografts of non-small cell lung cancer and melanoma**

Danfeng Cao^1,2^, Jie Yang^1^, Youchao Deng^1^, Meng Su^1^, Yeji Wang^1^, Xueqiong Feng^1^, Yi Xiong^1^, Enhe Bai^1^, Yanwen Duan^1,2,3*^, and Yong Huang^1,2,3*^

Correspondence to: Yong Huang ( [jonghuang@csu.edu.cn](mailto:jonghuang@csu.edu.cn))

Yanwen Duan ([ywduan66@sina.com](mailto:ywduan66@sina.com))

**This PDF file includes:**

Materials and Methods

Tables S1 to S8

Figures S1 to S25

**Materials and Methods**

**Cell lines and cell culture**

The human non-small cell lung cancer (NSCLC) cell lines A549 and NCI-H1299, the normal human bronchial epithelial BEAS-2B cells and normal human colon epithelial cells NCM-460, as well as human malignant melanoma cells A375 were obtained from the Institute of Chinese Academy of Sciences (Shanghai, China). The cell lines used in our present work were authenticated by short tandem repeat profiling and verified to be free of mycoplasma infection. A549, NCI-H1299 and NCM-460 cells were cultured in RPMI-1640 medium (Gibco, Grand Island, NY, USA). A375 and BEAS-2B cells were cultured in DMEM medium (Gibco, Grand Island, NY, USA). All cell lines were supplemented with 10% fetal bovine serum (FBS) (Gibco) unless otherwise noted, 1% L-glutamine, 100 U/ml penicillin and 100 μg/ml streptomycin, and cultured at 37℃ in a humidifier incubator with 5% CO_2_. Cells reaching to 70–80% confluency were subjected to different treatments.

**Anti-proliferative activity of tested compounds**

The anti-proliferative activities of platensimycin derivatives against A549 and NCI-H1299 tumor cell lines were evaluated using the Cell Counting Kit-8 assay (Kaiji, Nanjing, China). Briefly, A549 and NCI-H1299 cells were plated at a density of 2 × 10^3^ cells/well in 96-well plates (Thermofisher Scientific) overnight and subsequently treated with ~50 μM platensimycin derivatives or vehicle (0.1% DMSO) for 72 h. CCK-8 (10 μl) was next added into each well and the plate was incubated for 1 h at 37 °C. The OD_450_ value was measured by a microplate reader (TECAN, Spark 10M, Switzerland) for anti-proliferative activity analysis. For the cell viability assay, the tumor cell lines A549, NCI-H1299, and A375 and normal cell lines BEAS-2B and NCM-460 were seeded in 96-well plates at the density of 2×10^3^～2×10^5^ cells per well for 24 h to adhere and exposed to various concentrations of platensimycin, **6p**, 6-(4-bromophenyl)-platensic acid, TVB-3166, orlistat, cerulenin, or vehicle (0.1% DMSO) for 72 h. The IC_50_ determination was performed using a nonlinear regression curve-fitting algorithm by GraphPad Prism 5.0 software. Each group comprised six duplicated wells, and the assays were performed three times independently.

**Cell morphological observation by inverted microscope**

A549 and NCI-H1299 cells were seeded in 24-well plates at 20% density and cultured overnight. Then, these cells were treated with vehicle (0.1% DMSO), 10, 25 or 50 μM **6p** in 10% FBS containing medium for 48 h. Alteration of morphology in treated cells was observed under an inverted microscope (Leica, DFC450C, Wetzlar, Germany).

**Clonogenic assay**

A549 and NCI-H1299 were plated in 6-well plates at the density of 500 cells per well. After 24 h, cells were treated with vehicle (0.1% DMSO), 5 μM **6p**, 1 μM cis-platinum (CDDP) or the combination of **6p** and CDDP in 10% FBS containing medium for 10 days. Medium was gently removed at the end of 10^th^ day, and colonies were next fixed in fixing solution (4% paraformaldehyde) for 30 min at RT and stained with 0.1% crystal violet. The plates were carefully washed to remove excess of staining and dried overnight. The number of colonies that contained at least 10 cells was counted in triplicate dishes. Clonogenic ability was calculated by the formula: (the number of clones/the number of inoculated cells) ×100%.

**Flow-cytometric analysis of cell cycle**

A549 and NCI-H1299 cells (1 × 10^5^ per well) were seeded into a 6-well plate for 24 h to adhere at 37 °C, which were then exposed to various concentrations of **6p** (10, 25, and 50 μM) or vehicle (0.1% DMSO) for 48 h. Cells were harvested and washed with phosphate‐buffered saline (PBS) and fixed with 70% (v/v) alcohol for at least 24 h. Then cells were centrifuged and washed twice with PBS, followed by staining with propidium iodide (PI) dye containing RNase for 30 min in dark conditions at 37 ℃. The treated cells were analyzed by a flow cytometry (BD FACSCanto™ II; BD Biosciences, Franklin Lakes, NJ, USA) and the resulting data were analyzed by the Modfit software.

**Flow-cytometric analysis of Annexin V/PI binding**

A549 and NCI-H1299 cells were cultured at a density of 1 × 10^5^ cells/well in 6-well plates for 24 h, then treated with various concentrations of **6p** or vehicle (0.1% DMSO) for 48 h. The cells were centrifuged and washed three times with PBS. The supernatant was discarded and resuspended in 0.5 ml of cold PBS. The cells were subsequently stained using an Annexin V-FITC/PI Apoptosis Detection Kit (BD Bioscience) and a propidium iodide (PI) solution according to the manufacturer’s instructions. The labeled cells were analyzed in a flow cytometer (BD FACSCanto™ II; BD Biosciences, Franklin Lakes, NJ, USA). Data were analyzed by the FlowJo software.

**Wound healing, transwell migration and invasion assay**

Cell motility was measured using a wound healing analysis. Briefly, A549 and NCI-H1299 cells were grown into a confluent monolayer in a six‐well tissue culture dish. Cell monolayers were “wounded” using a P200 micropipette tip. The wounded monolayers were then washed twice with PBS to remove cell debris and incubated in cultured medium (RPMI-1640 medium and 1% FBS) with vehicle (0.1% DMSO) or **6p** (10 and 50 μM). The migrating cells in the denuded areas were recorded under an inverted microscope equipped with a camera for 24 h. Alternatively, in vitro cell migration or invasion assays were performed using a 24-well Boyden chamber precoated with Matrigel (BD Biosciences, San Jose, CA). Briefly, A549 and NCI-H1299 cells (1 × 10^5^ cells/well) were seeded at the inside of the precoated upper chambers and then treated with vehicle (0.1% DMSO) or **6p** (10 and 50 μM). The lower chamber was filled with RPMI-1640 medium containing 10% FBS. After incubation for 48 h, the migrated or invaded cells were fixed in paraformaldehyde for 30 min and stained with crystal violet. The migrated or invaded cells were counted under a microscope. The experiment was performed in triplicate.

**Cell growth rescue by exogenous free fatty acids.**

Frist, A549 and NCI-H1299 cells (4 × 10^3^ cells/well) were seeded in 96 well plates. After 24 h, **6p** was diluted in medium containing 1% FBS into various concentration gradients with TVB-3166 and orlistat as positive controls for 72 h. The IC_50_ values were calculated using OD_450_ value, which was measured by the microplate reader. Then, oleic acid (OA) (Sangon, Shanghai, China) and palmitic acid (PA) (Sigma-Aldrich, Shanghai, China) at a ratio of 2:1 were mixed with fatty acid-free bovine serum albumin (BSA) (Bomei, Hefei, China) to rescue the cell growth inhibition of **6p**. A549 and NCI-H1299 cells (4 × 10^3^ cells/well) were seeded in 96 well plates. After 24 h, cells were treated with various concentrations of **6p** and palmitate or **6p** and free fatty acid (oleic acid: palmitic acid = 2:1). Meanwhile, the control cells were treated with 1% BSA only. Each group comprised six duplicated wells, the cell viability was calculated by the OD_450_ value.

To calculate the Combination Indexes of **6p** and CDDP, a modified Bürgi’s formula shown in the following was used ^[1]^:


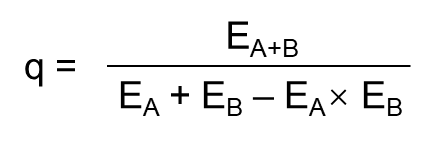


where EA, EB and EA+B are the inhibition efficiency of drug A, drug B and drug combination, respectively. “q < 1” suggests antagonism; “q > 1” suggests synergism. “q = 1” suggests simple addition. A tolerance of ± 0.15 was considered as an upper and lower limit.”

**Molecular docking and molecular dynamics simulation**

Docking studies were performed on an Intel Core i7 3.6 GHz processor, 4 GB memory with Windows 10 operating system using Molecular Operating Environment (MOE 2014.09, Chemical Computing Group, Canada) as the computational platform. All the energy minimizations were performed with MOE until a root mean square deviation (RMSD) gradient of 0.05 Kcal mol ^−1^ Å ^−1^ with an MMFF94X force field and the partial charges were automatically calculated. The crystallographic structure of human fatty acid synthase (FASN) KS-MAT didomain (PDB ID: 3HHD) was obtained from the Protein Data Bank. The enzyme was prepared for docking studies where (1) errors in crystallographic structure were corrected with prepare structure step and water molecules were removed from the complex; (2) hydrogen atoms and partial charges were added with Protonate 3D step.

The molecular dynamics studies were simulated on Intel Core i7 3.6 GHz processors, 128 GB memory, with Ubuntu 20.04 operating system. First, Gaussian 16 was used to fit the RESP charge of the compound. The protein was treated with Amber's ff14SB force field and the ligands (platensimycin and **6p**) were treated with Amber's GAFF force field. Using Amber tools to construct model and convert to GROMACS format using acpype. After equilibrium, we processed 100 ns production simulation and analyzed the generated MD trajectory file using GROMACS. Finally, the RMSD of protein skeleton and the non-bonding interaction energy between ligand and protein were calculated.

**RNA extraction and real-time qPCR**

Total RNA was extracted using TRIzol reagent (Invitrogen) according to the manufacturer’s procedure. The extracted RNA was reverse transcribed into cDNA by PrimeScript™ RT reagent Kit with gDNA Eraser (Perfect Real Time) (RR047A, Takara Bio, Japan). Real-time PCR was performed using double-stranded DNA dye SYBR Green (RR091A, Takara, Japan) and LightCycler 480 Roche system. PCR conditions were as follows: 30 s hot-start at 95°C followed by 45 cycles of 5 s at 95 °C, 30 s at 60 °C and 30 s at 72 °C; melting curve conditions were as follows: 5 s hot-start at 95 °C followed by 1 min at 65 °C, 1 s at 97 °C. Finally, 30 s cooling to 40 °C. All samples were analyzed in triplicate, and gene expression was normalized to *β*-actin mRNA levels. Primer sequences designed to detect specific genes are listed in supplementary Table S3.

**Protein isolation and western blot analysis**

Cell lysates were prepared in RIPA lysis buffer with fresh protease and phosphatase inhibitors mixture. Protein concentrations were measured by BCA protein assay reagent (P0006, Beyotime Biotechnology, China). In brief, protein (20 μg) was resolved by sodium dodecyl sulfate–polyacrylamide gel electrophoresis and transferred to polyvinylidene fluoride membranes. 6% or 8% polyacrylamide was used. After blocking in tris-buffered saline tween (TBST) buffer with 5% nonfat milk for 1 h, the membranes were incubated with the following primary antibodies: fatty acid synthase (FASN) (rabbit, 273 KDa, ab128870, 1: 30000 dilution, Abcam), sterol regulatory element binding protein (SREBP1) (mouse, 125 KDa and 65 KDa, ab3259, 1: 10000 dilution, Abcam), p-AKT^Ser473^ (rabbit, 60 KDa, 9271T, 1: 1000 dilution, CST), AKT (rabbit, 60 KDa, 9272S, 1: 1000 dilution, CST), p-mTOR^Sser2448^ (rabbit, 280 KDa, AM0223, 1: 1000 dilution, ABZOOM), mTOR (rabbit, 268 KDa, YT2913, 1: 1000 dilution, Immunoway), *β*-actin (mouse, 43 KDa, BS6007M, 1: 10000 dilution, Bioworld). After overnight incubation at 4 °C, the membranes were first washed in TBST buffer three times and then further incubated with secondary antibodies (rabbit, BS13278, 1: 10000 dilution, Bioworld; mouse, BS12478, 1: 10000 dilution, Bioworld). Immunoreactive bands were visualized by the ChemiDoc XRS+ imaging system (Bio-Rad, USA). For quantification of the protein bands, we utilized Image J software (National Institutes of Health, USA) to obtain densitometric values.

**Immunofluorescence analysis**

NCI-H1299 cells were seeded in a 35-mm dish at a density of 2 × 10^4^ cells/mL. After 24 h, cells were treated with 25 μM **6p** for 48 h. Then cells were fixed in 4% paraformaldehyde for 30 min, permeabilized in 0.2% Triton X-100 for 15 min, and blocked in donkey serum (SL034, Solarbio, China) for 30 min at room temperature. Cells were then incubated with primary antibody (FASN, rabbit, ab128870, 1: 250 dilution, Abcam) in the blocking buffer at 4 °C overnight. PBS was used to replace the primary antibody in negative controls. After washing three times with PBS, the cells were incubated with secondary antibody. The secondary antibody was donkey anti-rabbit Alexa Fluor 488 (A-21206, 1: 100 dilution, ThermoFisher Scientific), and was used for 45 min at room temperature. DAPI (S2110, Solarbio, China) was applied to stain the cell nucleus for 15 min. Immunofluorescence-stained cells were examined under a laser-scanning confocal microscope (Nikon, Japan).

**RNA sequencing and data analysis**

To characterize the global transcriptional effect and changes in gene associated with *de novo* lipogenesis, NCI-H1299 cells were seeded in 6-well plates at a density of 8×10^4^ cells/well. The following day, the treatment media with vehicle (0.1% DMSO) or **6p** (25 μM) were added and cells were incubated for an additional 48 h. All treatments were performed in triplicate. For RNA sequencing, cells were lysed using TRIzol reagent (9108, TaKaRa, China), and then sequenced on an Illumina platform (Shanghai OE Biotech Co., Ltd.). Briefly, the total RNA was extracted and purified. Then the purified RNA was reversely transcribed into cDNA for library construction. Sequencing data were collected on the Illumina platform, and paired-end reads were generated. Raw data were processed using Trimmomatic software. The reads containing poly-N, low-quality reads and bases were removed to obtain the clean reads. Then, the clean reads were mapped to the reference genome using hisat2 software. The fragments per kilobase of transcript per million mapped reads value of each gene were calculated using cufflinks software, and the read counts of each gene were obtained by htseq-count software. Differentially expressed genes were identified using the DESeq software and fold change (FC) was calculated. The significance of the difference (*p* values) was tested using nbinom test. Finally, genes with significant changes in mRNA abundance were identified using the statistical (p < 0.05) and (FC > 1.5-fold) criteria. Gene ontology enrichment and Kyoto Encyclopedia of Genes and Genomes pathway enrichment analysis of differentially expressed genes were respectively performed using hypergeometric distribution. The RNA sequencing data have been deposited in the Gene Expression Omnibus with accession no. GSE198816.

**Lipidomics sample preparation and lipid profiling**

NCI-H1299 cells were seeded in 100-mm dishes at a density of 15 × 10^4^ cells/ml in RPMI-1640 medium with 10% FBS. After overnight incubation, the cells were treated with 25 μM **6p** and 25 μM TVB-3166 for 48 h, washed with ice-cold PBS, and flash-frozen at -80℃. Five biological replicates were set for each group (five vehicles and five treated with 25 μM **6p** and TVB-3166). Dry pellets of 5 × 10 ^6^ cells in dry-ice were sent to Shanghai Major Bio Co., Ltd. for lipid extraction and LC-MS/MS analysis. As a part of the system conditioning and quality control process, a pooled quality control sample (QC) was prepared by mixing equal volumes of all samples. QC samples were inserted into the analysis queue to evaluate the system stability and data reliability during the whole experimental process.

After UPLC-MS analyses, the raw data were imported into the LipidSearch (Thermo, CA) for peak detection, alignment and identification. The lipids were identified by MS/MS fragments. The preprocessing results generated a data matrix that consisted of the lipid class, retention time (RT), mass-to-charge ratio (m/z) values, and peak intensity. Lipidomic features detected at least 80% in any set of samples were retained. After filtering, minimum lipid values were input for specific samples in which the lipid levels fell below the lower limit of quantitation and each lipid feature was normalized by sum. Features with the relative standard deviation (RSD) of QC > 30% were discarded. Following normalization procedures and imputation, statistical analysis was performed on log transformed data to identify significant differences in metabolite levels between comparable groups.

A multivariate statistical analysis was performed using ropls (*R* package) from Bioconductor on Majorbio Cloud Platform (https://cloud.majorbio.com). Principle component analysis (PCA) using an unsupervised method was applied to obtain an overview of the lipidomic data, general clustering, trends, or outliers were visualized. Orthogonal partial least squares discriminate analysis (OPLS-DA) was used for statistical analysis to determine global lipidomic changes between comparable groups. The model validity was evaluated from model parameters R^2^ and Q^2^, which provide information for the interpretability and predictability of the model, respectively, and avoid the risk of over-fitting. Variable importance in the projection (VIP) was calculated in OPLS-DA model. The *p* values were estimated with paired Student’s t-test on Single dimensional statistical analysis. Statistically significant among groups were selected with VIP > 1 and *p* < 0.05. Then differential lipids were mapped into their biochemical pathways through metabolic enrichment and pathway analysis based on database search (KEGG, <http://www.genome.jp/kegg/>). These lipids can be classified according to the pathways they are involved or the functions they perform. Enrichment analysis was usually to analyze a group of metabolites in a function node whether appears or not. The principle was that the annotation analysis of a single metabolite develops into an annotation analysis of a group of metabolites. Scipy.stats (Python packages) (<https://docs.scipy.org/doc/scipy/>) was exploited to identify statistically significantly enriched pathway using Fisher’s exact test.

**Animals**

Five-week-old female BALB/c nude mice weighing 20.0 ± 2.0 g were obtained from Hunan Slack Jingda Experimental Animal Co., Ltd. (Changsha, China). All mice were housed in an SPF-level animal room under a 12 h light/dark cycle with an ambient temperature of 26 °C and relative humidity of 50%. Mice had free access to food and water. All animal experiments were conducted in accordance with guides and protocols approved by the Institutional Animal Care and Treatment Committee of Central South University. The Animal Ethics Committee of Central South University approved the animal experiments (No. 2019sydw0178).

**Determination of 6p efficacy in vivo**

Animals were acclimatized for 5−6 days prior to initiating the study. A549 cells (5 × 10^6^ cells) were injected with matrigel (1: 1) into the right armpits of BALB/c nude mice to establish the subcutaneous tumor-bearing model. When the tumor reached a volume of 100 mm^3^, mice were randomly divided into 5 groups for treatment (n = 5), which included control (PBS), positive control orlistat (240 mg/kg), CDDP (5 mg/kg), **6p** (50 mg/kg), and CDDP + **6p**. Orlistat and CDDP were administered intraperitoneally every other day as described in references. CDDP was discontinued after administration for 6 times. PBS and **6p** were injected adjacent to the tumor through subcutaneous administration every other day. The length and width of tumor as well as the body weight of each mouse were measured daily from the time of administration. The volume (V) of tumor was calculated by the formula V = 1/2 × length × width^2^. After 29 days, mice were euthanized to collect tumors, liver, and kidney tissues. The tumor samples were weighted for final weights and picture, then were used to examine the expression of FASN and p-Akt through western blot assays and immunohistochemistry.

A375 cells (5 × 10^6^ cells) were injected with matrigel (1: 1) into the right armpits of BALB/c nude mice to establish the subcutaneous tumor-bearing model. When the tumor reached a volume of 100 mm^3^, mice were randomly divided into 8 groups for treatment (n = 5). Among them, 4 groups were used to further test the anti-tumor effect of **6p** in vivo compared with the controls, which included control (PBS), positive control orlistat (240 mg/kg), positive control PTM (50 mg/kg), and **6p** (50 mg/kg). All agents were administered once every two days for 6 times. The length and width of tumor as well as the body weight of each mouse were measured daily from the time of administration. The other four groups were used to preliminarily evaluate the pharmacokinetic characteristics of **6p** in vivo. This part included control (PBS, i.v.), positive control orlistat (240 mg/kg, i.p.), **6p** (50 mg/kg, i.v.), and **6p** (50 mg/kg, s.c.), all agents were administered every other day for 6 times. The volume (V) of tumor was calculated by the formula V = 1/2 × length × width^2^. After 12 days, mice were euthanized to collect blood, liver, kidney, and tumor tissue. The tumor tissues were pictured and weighed for final weights. The blood samples were analyzed for inflammatory response by Wuhan Servicebio Technology Co., Ltd. (Hubei, China).

**Immunohistochemistry (IHC)**

At the endpoint of animal monitoring, the mice were euthanized for pathological studies. For IHC staining, the tumor tissues were quickly removed and fixed in a 4% paraformaldehyde solution for 24 h at 4 °C and then embedded in paraffin. The paraffin-embedded tissues were sectioned at 4 µm and mounted on precoated glass slides. The following primary antibodies were used: p-AKT^Ser473^ (rabbit, AF300996, 1: 200 dilution, AiFang biological), Ki-67 (mouse, AF20068, 1:150 dilution, AiFang biological). All photographs were captured with an inverted fluorescence microscope (DMIL LED/DFC7000T, Leica).

**Statistical analysis**

All experiments were carried out for at least three independent replicates. Statistical analysis was performed and calculated using GraphPad Prism 9.0 software. The normal distribution of the data was evaluated with Shapiro-Wilk test. Student’s t-tests were used to determine significant differences between two groups. One-way ANOVA followed by Tukey’s tests were used for experiments involving more than two groups. Two-way ANOVA followed by Sidak’s tests were used to correct multiple comparisons when two independent variables were tested. All data were presented as the mean ± SD. *P* values of less than 0.05 were considered statistically significant.

In the current study, statistical method was employed to determine the sample size according to our previously published data in the A375 nude xenograft tumor model ^[2]^. Three mice for each group were required to detect a 53.77% difference between the treatment and model group in the analysis of tumor volume (or tumor weight) with an 100% power and a type Ι error rate of 5%. Therefore, at least three animals for each group have been used in our present study.

**Table S1.** Comparison of fatty acid synthase (FASN) inhibitors in FASN expression, RNA-seq, and lipid profile.

| **FASN inhibitors** | **Cell lines** | **Culture**  **conditions** | **Dosage/treatment time** | **FASN expression** | **RNA-seq (Genes involved**  **in de novo lipid synthesis)** | **Lipid profile/**  **Lipid droplets** | **Ref** |
| --- | --- | --- | --- | --- | --- | --- | --- |
| TVB-3166  (KR) | CALU-6;  COLO-205;  OVCAR-8;  22RV-1;  PANC-1; | Advanced MEM, 1% charcoal filtered FBS | 0.02, 0.2, or 2 μM, 72 h for WB  0.1, 1 μM/48 h, 72 h for RNA-seq | Unchanged/**up** | **up** | **—** | [3] |
| TVB-3664  （KR） | MHCC97H/;  HLE;  murine sgPTEN/c-MET HCCs; | 10% FBS | 10 mg/kg/day, 3 weeks for RNA-seq, WB and Oil Red O dye staining | Unchanged | **—** | **Down** | [4] |
| Fasnall  (Co-factor) | MCF7;  MDA-MB-468;  BT474;  SKBR3;  BT474; | 10% FBS | 10 μM, 24h for WB  10 μM, 2h for lipid profile | Unchanged | **—** | **up** | [5] |
| IPI-9119  (TE) | LNCaP;  22Rv1;  LNCaP-95; | 10% FBS | 0.05, 0.1, 0.25,0.5 μM, 6 days for WB  0.1, 0.5 μM, 6 days for RNA-seq  0.1, 0.5 μM, 6 days for lipid profile | **up** | **up** | **Down** | [6] |
| Orlistat  (TE) | Hep3B | 5% FBS | 3, 10, 30 μM, 24h for WB  10 μM, 24h for Oil Red O dey staning | **Down** | **—** | **Down** | [7] |
| Cerulenin  (KS) | HCT116 | 10% FBS | 100 μM, 24 h for WB | **Down** | **—** | **—** | [8] |

**Table S2.** IC_50_ values of FASN inhibitors TVB-3166, orlistat, cerulenin, platensimycin, and **6p** against A549, NCI-H1299, A375, BEAS-2B, and NCM-460 cell lines.

| **Compounds** | **IC_50_（μM）** | | | | |
| --- | --- | --- | --- | --- | --- |
|  | **A549** | **NCI-H1299** | **A375** | **BEAS-2B** | **NCM-460** |
| Platensimycin | ＞500 | ＞500 | ＞500 | ＞500 | ＞500 |
| TVB-3166 | 23.95±1.32 | 62.89±0.45 | 49.82±0.15 | 78.84±1.70 | 54.56±0.49 |
| Orlistat | 43.15±1.01 | 34.86±3.97 | 40.80±0.72 | 16.19±1.95 | 32.85±1.27 |
| Cerulenin | 25.82±1.75 | 19.11±0.09 | 16.24±0.06 | 17.90±4.16 | 16.24±0.04 |
| **6p** | 16.95±1.96 | 24.62±1.33 | 22.05±1.98 | 54.50±1.78 | 41.31±0.14 |

**Table S3.** Primers used for real-time quantitative PCR.

| Gene | Sequence (5ʹ－3ʹ) |
| --- | --- |
| *FASN* | Sense: AAGGACCTGTCTAGGTTTGATGC |
|  | Antisense: TGGCTTCATAGGTGACTTCCA |
| *ACC* | Sense: ATGTCTGGCTTGCACCTAGTA |
|  | Antisense: CCCCAAAGCGAGTAACAAATTCT |
| *SREBF1* | Sense: ACAGTGACTTCCCTGGCCTAT |
|  | Antisense: GCATGGACGGGTACATCTTCAA |
| *SCD1* | Sense: TCTAGCTCCTATACCACCACCA |
|  | Antisense: TCGTCTCCAACTTATCTCCTCC |
| *β-actin* | Sense: CATGTACGTTGCTATCCAGGC  Antisense: CTCCTTAATGTCACGCACGAT |

**Table S4.** Validation of the OPLS-DA model.

| **Model** | **A** | **N** | **R^2^X (cum)** | **R^2^Y (cum)** | **Q^2^ (cum)** |
| --- | --- | --- | --- | --- | --- |
| TVB3166/Control | 1+1+0 | 10 | 0.853 | 1 | 0.998 |
| **6p**/Control | 1+1+0 | 10 | 0.662 | 1 | 0.990 |
| TVB-3166/**6p** | 1+1+0 | 10 | 0.790 | 1 | 0.995 |

**Table S5.** The top 20 glycerolipids according to the VIP value, detected in **6p**-treated and control groups, in which red color label stands for increased glycerolipids and green color label stands for decreased glycerolipids.

| **Metabolite** | **RT (min)** | **VIP** | **Fold change** | **P-value** |
| --- | --- | --- | --- | --- |
| TG (16:0/20:4/22:6) | 12.1937 | 2.174935 | 4.096594 | 4.59E-05 |
| MGDG (16:1/18:1) | 6.872432 | 2.156998 | 0.22393 | 0.000112 |
| TG (15:0/14:0/15:0) | 12.275 | 2.069702 | 3.465432 | 0.000119 |
| TG (18:1/22:5/22:5) | 12.458 | 1.962903 | 3.015376 | 6.76E-05 |
| TG (20:0/22:5/22:5) | 13.42879 | 1.957311 | 2.885159 | 4.24E-07 |
| TG (20:3/22:3/22:3) | 13.81439 | 1.933264 | 3.051906 | 0.004969 |
| TG (16:1/14:1/18:1) | 11.91956 | 1.891492 | 0.349387 | 0.001467 |
| TG (16:0/17:1/22:6) | 12.57524 | 1.861373 | 2.803666 | 6.24E-05 |
| TG (18:1/22:5/22:6) | 12.23729 | 1.85318 | 2.792797 | 0.000199 |
| TG (18:1/22:4/22:6) | 12.69486 | 1.851395 | 2.571838 | 3.68E-08 |
| TG (16:0/22:6/22:6) | 12.00486 | 1.850886 | 2.567514 | 2.12E-07 |
| DGDG (16:0/16:1) | 6.549322 | 1.849927 | 0.325645 | 0.000308 |
| MGDG (16:0e/16:0) | 8.207198 | 1.823697 | 0.352729 | 4.36E-10 |
| MGDG (16:0/18:1) | 7.656892 | 1.822169 | 0.354713 | 1.73E-13 |
| TG (22:1/22:5/22:5) | 13.45059 | 1.808674 | 2.452633 | 1.31E-08 |
| TG (18:1/22:6/22:6) | 11.99938 | 1.796077 | 2.536105 | 3.30E-05 |
| TG (18:1/20:4/22:6) | 12.23108 | 1.794545 | 2.443611 | 2.53E-06 |
| DG (16:0/16:0) | 8.612 | 1.779949 | 0.414463 | 2.70E-07 |
| TG (18:1/17:1/22:6) | 12.56416 | 1.762948 | 2.353993 | 3.09E-08 |
| TG (18:1/22:4/22:5) | 12.96558 | 1.75819 | 2.445806 | 2.20E-05 |

**Table S6.** The top 20 glycerophospholipids according to the VIP value, detected in **6p**-treated and control groups, in which red color label stands for increased glycerophospholipids.

| **Metabolite** | **RT (min)** | **VIP** | **Fold change** | **P-value** |
| --- | --- | --- | --- | --- |
| PC (20:1/22:4) | 8.375369 | 2.756084 | 7.032706 | 0.001106 |
| PC (18:2e/18:0) | 8.972527 | 2.741541 | 8.199794 | 2.80E-06 |
| PI (17:1/20:4) | 5.59 | 2.558394 | 8.380517 | 5.78E-05 |
| PC (20:5/20:4) | 5.498579 | 2.544149 | 7.470444 | 4.31E-07 |
| DLCL (22:5/15:0) | 5.057 | 2.513912 | 7.589054 | 8.74E-05 |
| PC (22:5/20:4) | 6.111276 | 2.462246 | 5.829338 | 2.10E-05 |
| MePC (18:1/20:4) | 6.287765 | 2.390419 | 4.844316 | 5.43E-05 |
| PI (22:5/20:4) | 5.373476 | 2.384171 | 7.435342 | 7.10E-05 |
| CL (22:6/20:4/22:1/22:6) | 5.81 | 2.337552 | 5.605626 | 0.0001022 |
| PE (20:5/20:4) | 5.517499 | 2.32449 | 5.552719 | 0.00135 |
| BisMePA (22:6/22:6) | 5.870784 | 2.313467 | 4.485199 | 9.92E-05 |
| PC (15:0/22:4) | 8.1112 | 2.271185 | 4.085956 | 4.75E-11 |
| PG (22:5/22:6) | 5.05239 | 2.200422 | 4.049041 | 2.27E-05 |
| PG (18:0/20:4) | 6.815 | 2.198909 | 4.547112 | 2.76E-07 |
| PC (22:3/22:6) | 6.697393 | 2.133948 | 3.481423 | 5.76E-09 |
| MePC (22:6/21:0) | 7.274921 | 2.092694 | 3.338049 | 5.02E-07 |
| PI (19:0/20:4) | 5.794 | 2.060844 | 3.766797 | 3.49E-09 |
| PC (10:0e/20:4) | 5.129 | 2.050392 | 3.30274 | 3.59E-05 |
| PC (20:3/22:6) | 6.111945 | 2.03307 | 3.637751 | 6.88E-10 |
| PC (18:3/22:6) | 5.49454 | 2.029166 | 3.09136 | 1.33E-07 |

**Table S7.** The top 10 sphingolipids according to the VIP value, detected in **6p**-treated and control groups, in which red color label stands for increased sphingolipids and green color label stands for decreased sphingolipids.

| **Metabolite** | **RT (min)** | **VIP** | **Fold change** | **P-value** |
| --- | --- | --- | --- | --- |
| Cer (m18:0/16:0) | 7.80925 | 2.712376 | 0.134039 | 5.93E-11 |
| Cer (d18:0/26:1) | 10.797 | 2.575593 | 0.120006 | 4.05E-07 |
| Cer (m18:0/24:1) | 10.38567 | 2.552643 | 0.167252 | 3.74E-09 |
| Cer (m18:0/26:1) | 11.18335 | 2.30615 | 0.231912 | 1.72E-08 |
| SM (d17:0/18:0) | 7.21778 | 2.24447 | 4.611506 | 7.23E-05 |
| SPH (d18:1) | 1.577714 | 2.243629 | 0.264934 | 0.008815 |
| Cer (m18:0/24:0) | 11.3326 | 2.206595 | 0.262804 | 8.32E-09 |
| Cer (m18:0/22:0) | 10.50712 | 1.97596 | 0.342452 | 1.28E-08 |
| Cer (d18:0/24:0) | 10.94745 | 1.827302 | 0.384185 | 0.000176 |
| Cer (d18:0/16:0) | 7.338187 | 1.795782 | 0.337362 | 3.64E-05 |

**Table S8.** The analysis of blood samples from subcutaneous A375 tumor-bearing mice after treatment (collected at the 12^th^ day).

| **Groups** | **White blood cell counts (×10^9^/L)** | **Lymphocyte cell counts (×10^9^/L)** | **Monocyte cell counts (×10^9^/L)** | **Red blood**  **cell counts**  **(×10^12^/L)** | **Hemoglobin**  **(g/L)** |
| --- | --- | --- | --- | --- | --- |
| PBS | 12.50 ± 4.73 | 10.80 ± 4.24 | 0.20 ± 0.14 | 9.70 ± 0.49 | 154.50 ±4.95 |
| Orlistat | 12.17 ± 6.22 | 8.93 ± 6.14 | 0.60 ± 0.50 | 8.49 ± 0.57 | 130.67±11.15 |
| **6p** (i.v.) | 8.07 ± 6.79 | 5.47 ± 5.43 | 0.33 ± 0.32 | 9.88 ± 0.40 | 155.00 ±4.00 |
| **6p** (s.c.) | 6.87 ± 2.65 | 4.83 ± 2.41 | 0.17 ± 0.06 | 9.96 ± 0.56 | 155.33 ±8.02 |
| Reference range | 0.8 – 6.8 | 0.7 – 5.7 | 0.0 – 0.3 | 6.36 – 9.42 | 110 – 143 |

**Fig. S1.**

**Figure. S1.** Chemical structures of 6-acrylyl, alkynyl and cyclized platensimycin derivatives (A1-A32), which were prepared through Heck, Sonogashira or Sonogashira/ cycloaddition cascade reactions ^[9]^.

**Fig. S2.**

**Figure. S2.** Chemical structures of sulfur-containing platensimycin derivatives (B1-B11), which were prepared from platensimycin oxirane through the addition of various thiols ^[10]^.

**Fig. S3.**

**Figure. S3.** Chemical structures of sulfur-containing platensimycin derivatives (C1-C20), which were prepared through sulfa-Michael addition ^[11]^.

**Fig. S4.**

**Figure. S4.** Chemical structures of halogen-substituted platensimycin aminobenzoic acid analogues (D1-D14) ^[12]^.

**Fig. S5.**

**Figure. S5.** Chemical structures of platensimycin oxime, hydrazine derivatives (E1-E10), which were prepared through Schiff base formation on the terpene scaffold ^[13]^.

**Fig. S6.**

**Figure. S6.** Chemical structures of 6-aryl conjugated platensimycin derivatives (F1-F20), which were prepared through Suzuki-Miyaura cross-coupling reactions ^[14]^.

**Fig. S7.**


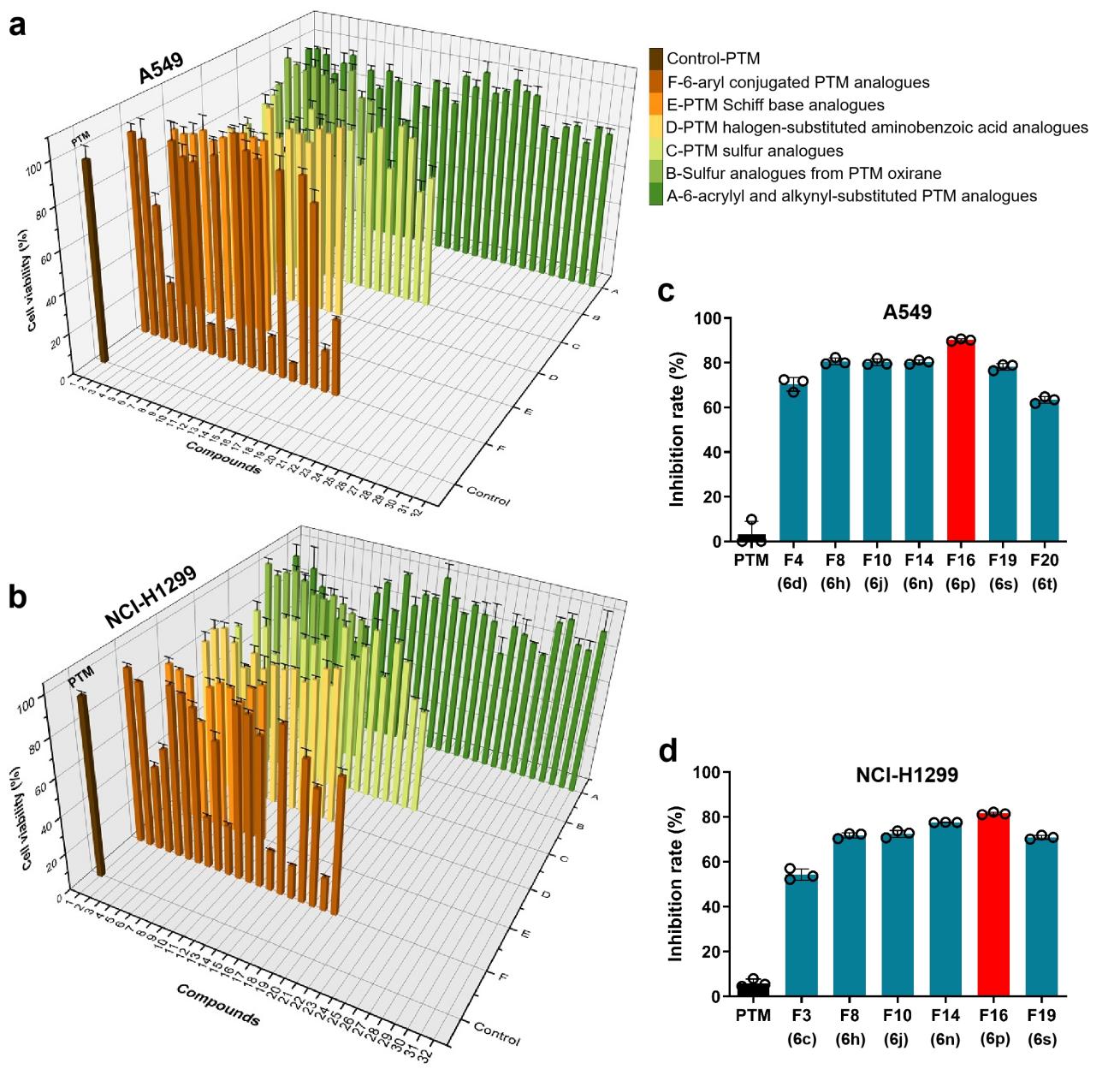


**Figure. S7. Anti-proliferative activities of** **platensimycin (PTM) derivatives against A549 and NCI-H1299 cell lines.** **a and b** The cytotoxic activity of 107 synthesized platensimycin derivatives was tested against A549 cells (**a**) and NCI-H1299 cells (**b**) with the CCK-8 kit. All of the platensimycin derivatives were applied at approximately 50 μM and platensimycin (50 μM) was used as a control. **c and d** The cell inhibition rate of promising compounds, including **6c**, **6h**, **6j**, **6n**, **6p**, and **6s** against A549 cells (**c**) and NCI-H1299 cells (**d**). Compound **6p** (red) has the highest inhibitory efficacy against both cancer cells and was thus selected for the next study.

**Fig. S8.**


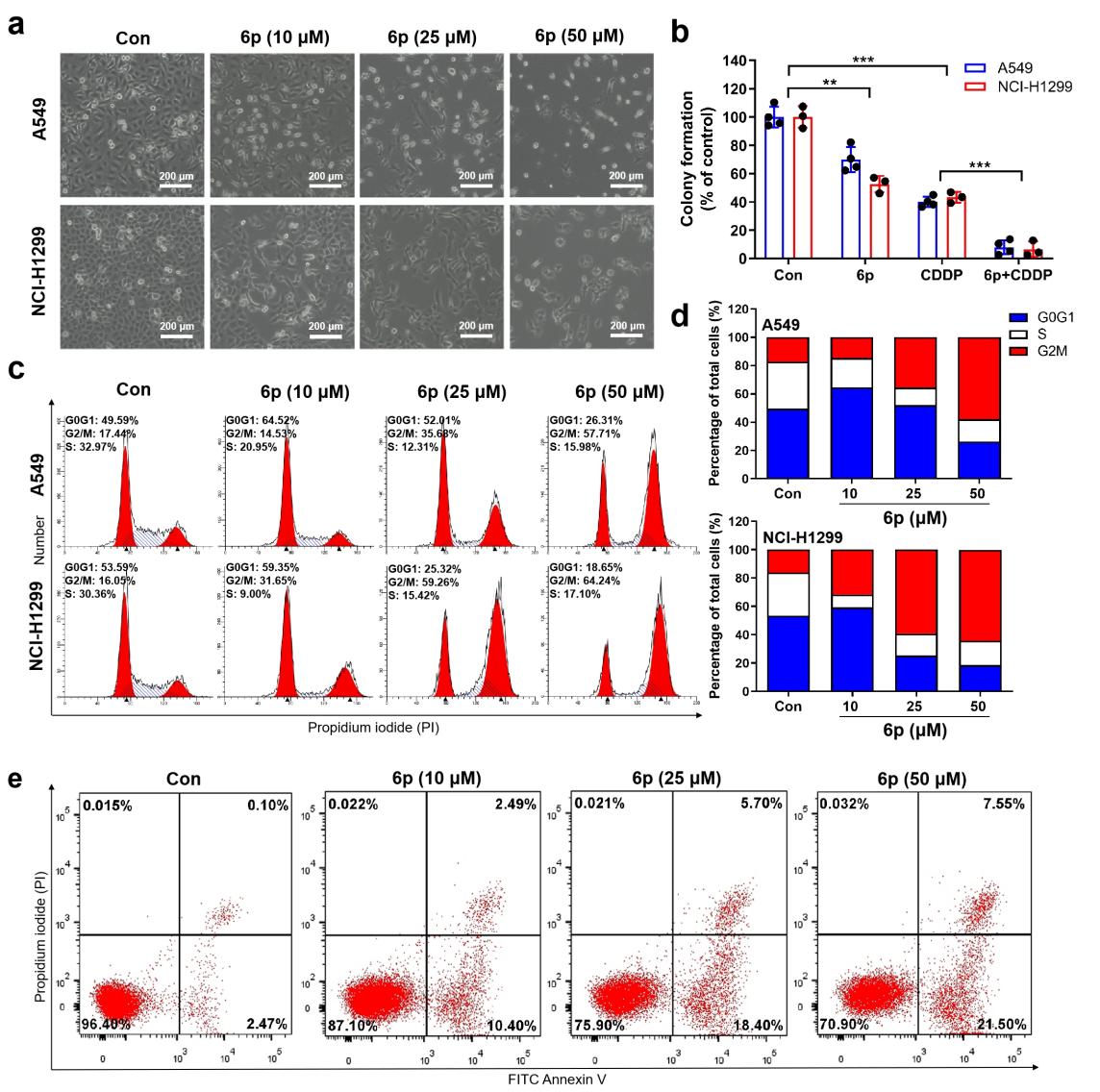


**Figure. S8. Compound 6p inhibits cell growth, blocks cell cycle, and induces cell apoptosis. a** The cell morphology of A549 and H1299 cells after **6p** treatment for 48 h in RPMI plus 10% FBS. **b** A549 and NCI-H1299 tumor cells were treated with 5 μM **6p** and 1 μM cis-platinum diaminedichloride (CDDP) and the inhibition of colony formation was assessed in the single-agent and combination group (n = 3), in which **6p** (5 μM) and CDDP (1 μM) showed apparent synergism against both A549 and NCI-H1299 cell lines with q values of 1.28 and 1.21, respectively ^[1]^. **c** After A549 and NCI-H1299 tumor cells were incubated with 10, 25 and 50 μM of **6p** for 48 h, the cell cycle distributions in G0/G1, S and G2/M phases were detected by flow cytometry using PI staining. **d** The percentage of cell populations in each phase of the cell cycle relative to the whole cell population (100%) was shown in histogram. **e** Cell apoptosis induced by **6p** through annexin V-FITC/PI staining. Data were shown as means ± SD, One-way ANOVA test. ***p* <0.01, ****p* <0.001.

**Fig. S9.**


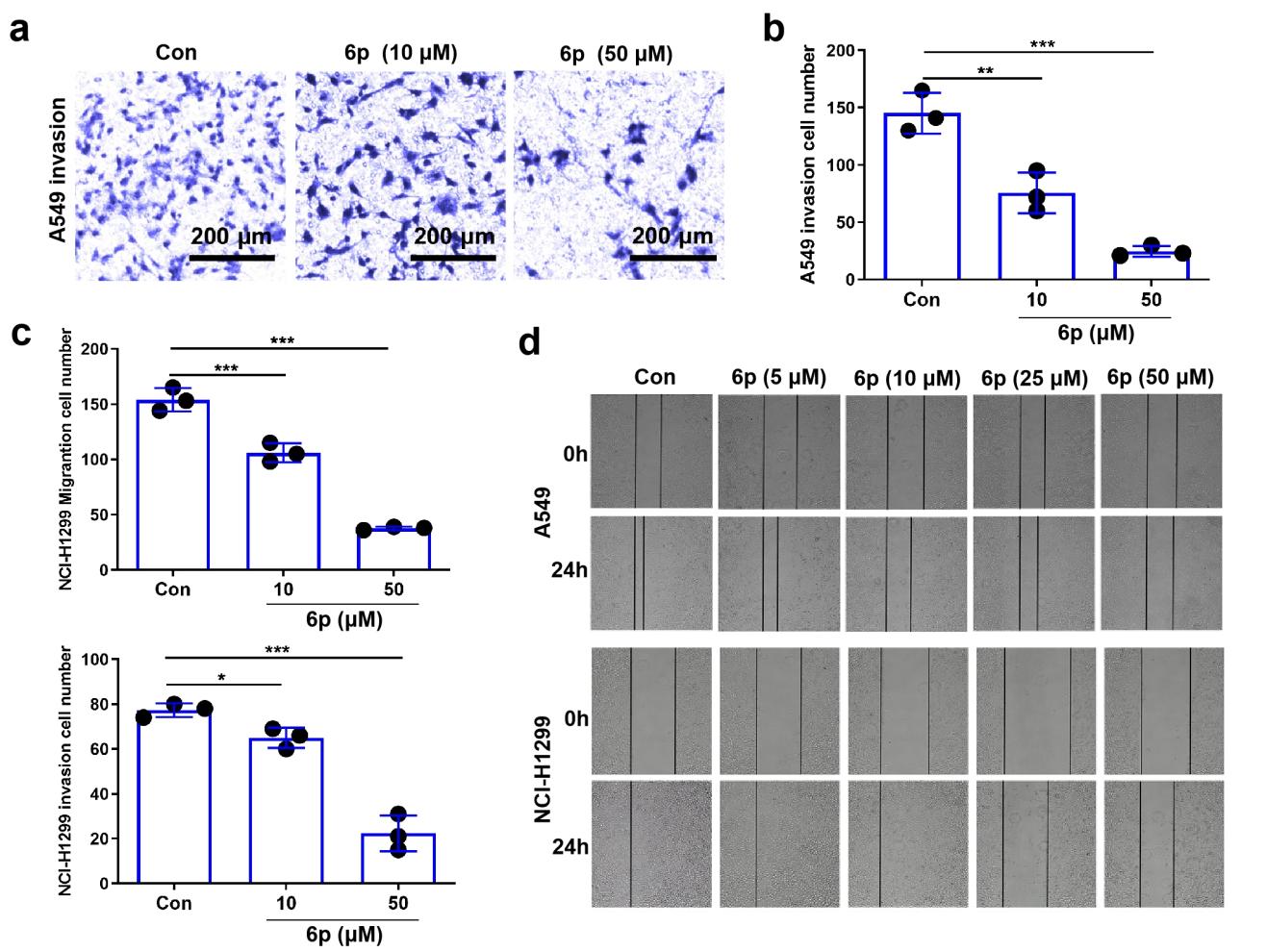


**Figure. S9. Compound 6p suppresses the migratory and invasive capability of treated tumor cells. a** The transwell assay was performed to determine the invasion in A549 cells treated with vehicle or **6p** (10 and 50 μM) for 48 h. **b** The number of invaded A549 cells was counted in histogram (n = 3). **c** The number of migration and invasion NCI-H1299 cells was counted in histogram (n = 3). **d** The wound healing assay was conducted to evaluate the effect of **6p** on the cell migratory ability in A549 and NCI-H1299 cells. Data were shown as means ± SD, One-way ANOVA test. **p* <0.05, ***p* <0.01, ****p* <0.001.

**Fig. S10.**


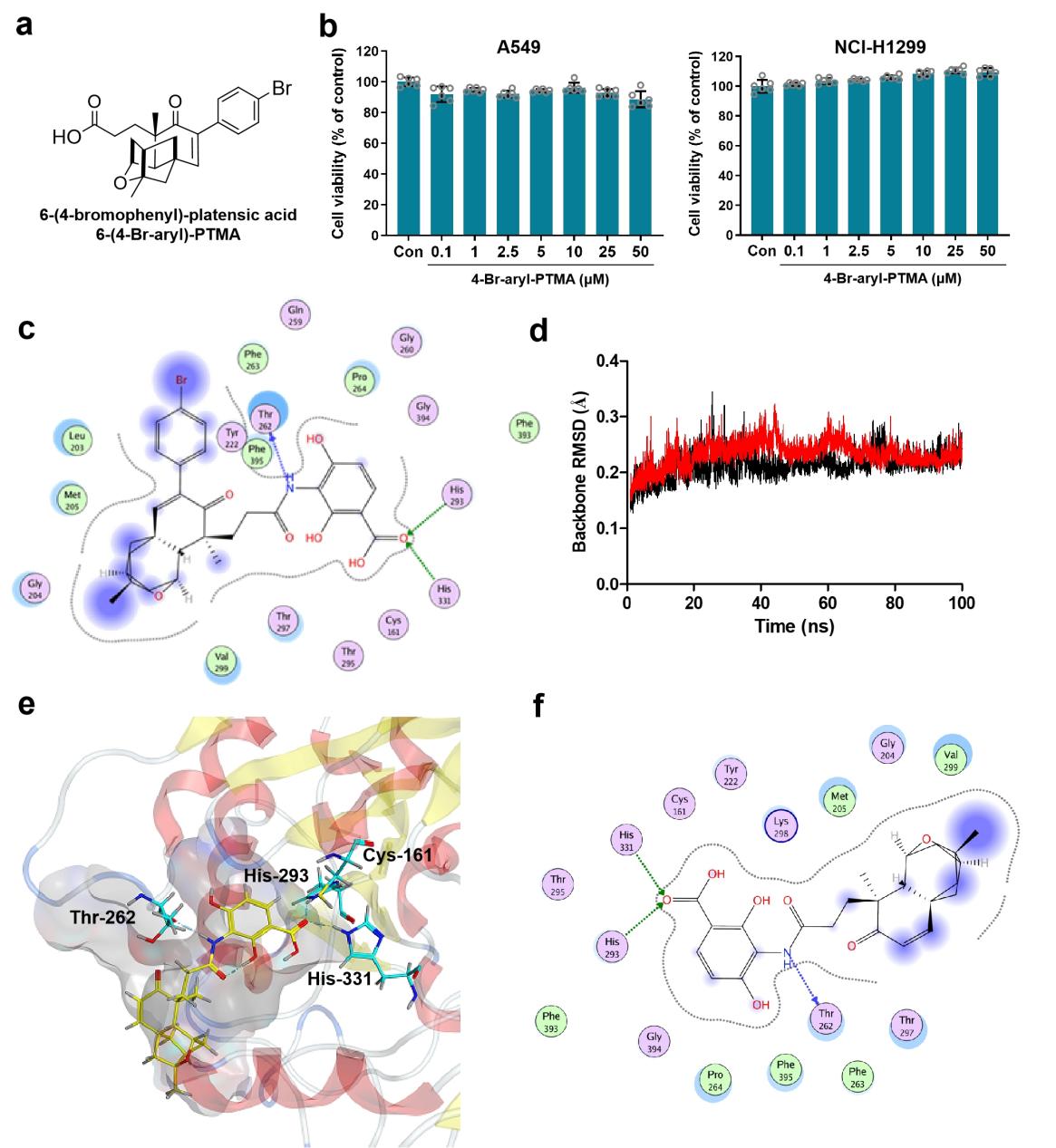


**Figure. S10. The cytotoxicity activity of 6-(4-bromophenyl)-platensic** **acid, as well as the docking and molecular dynamics simulations of 6p and platensimycin.** **a** The structure of 6-(4-bromophenyl)-platensic acid (PTMA) ^[14]^. **b** The cytotoxic activity of 6-(4-Br-aryl)-PTMA against A549 and NCI-H1299 cells (n = 6). **c** The binding mode of **6p** with human FASN KS-MAT Didomain (PDB ID: 3HHD) was shown in the 2D model, with interactions towards His_293_ and His_331_. **d** The root-mean-square deviation (RMSD) values of the human FASN KS-MAT backbone between protein and ligands (**6p**, red and platensimycin, black) in the complexes from 1 and 100 nanoseconds (ns). **e** The predicted docking mode of platensimycin (yellow) in human FASN KS-MAT didomain (3HHD). **f** The binding mode of platensimycin with human FASN KS-MAT Didomain (PDB ID: 3HHD) was shown in the 2D model.

**Fig. S11.**


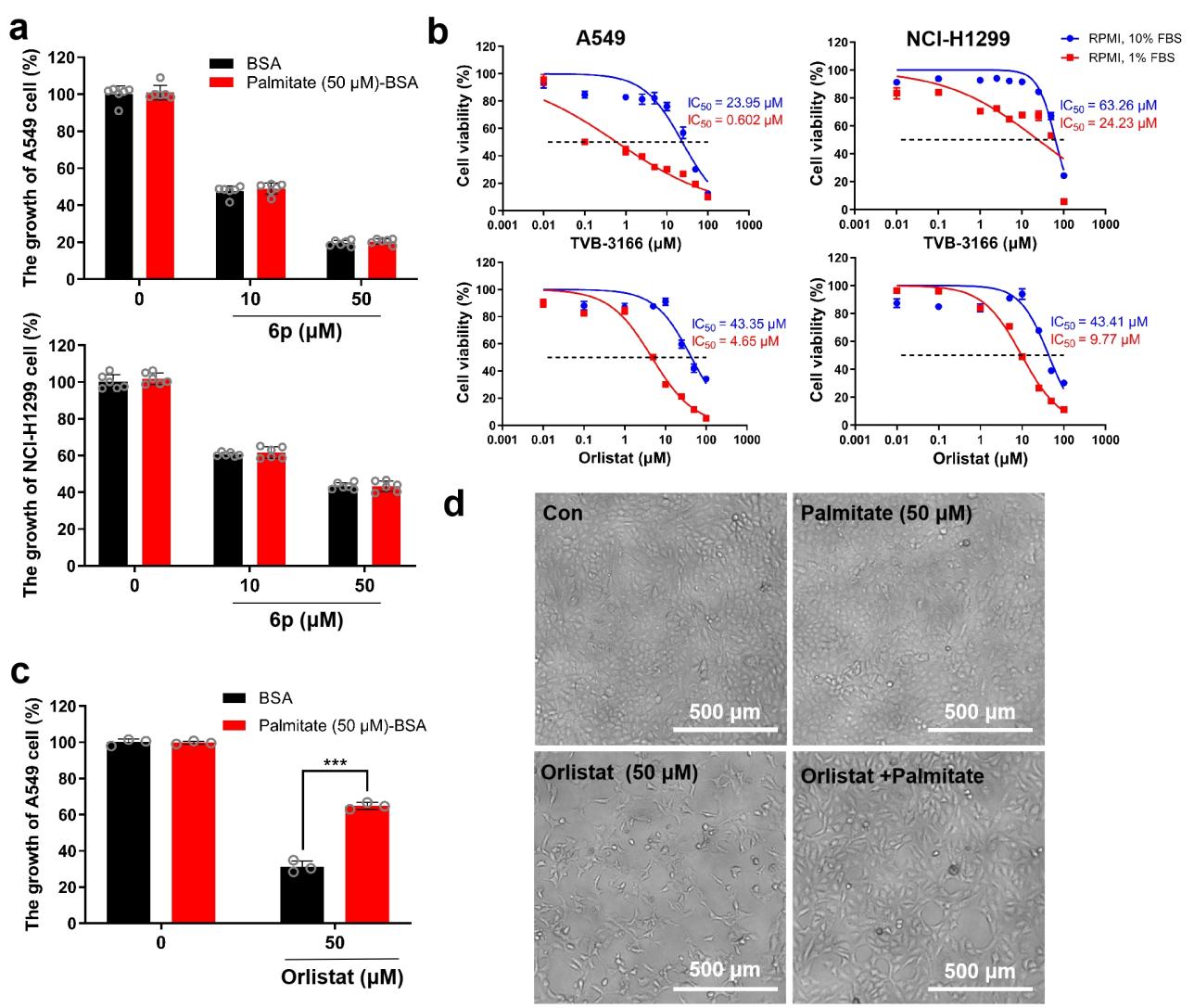


**Figure. S11. Exogenous FFAs (oleic acid: palmitate = 2:1) rather than palmitate rescues the cytotoxicity induced by 6p.** **a** Cell growth inhibition by **6p** was rescued by the addition of exogenous FFAs rather than palmitate in A549 and NCI-H1299 cells (n = 6). **b** The IC_50_ values of TVB-3166 and orlistat were reduced in culture medium with the decreased serum concentration (1% FBS) in A549 and NCI-H1299 cells. **c** Cell growth inhibition by orlistat was rescued by the addition of exogenous palmitate (n = 3). **d** Orlistat (50 μM) exposure caused cell death, while palmitate (50 μM) reversed the orlistat-induced cell damage in A549 cells. Orlistat is a clinically-used inhibitor of the thioesterase domain of FASN, while **6p** may interact with the ketosynthase domain of FASN. It is likely that the different modes of action of these two compounds led to this difference. Data were shown as means ± SD, Student’s t tests. ****p* <0.001 orlistat + palmitate versus orlistat group.

**Fig. S12.**


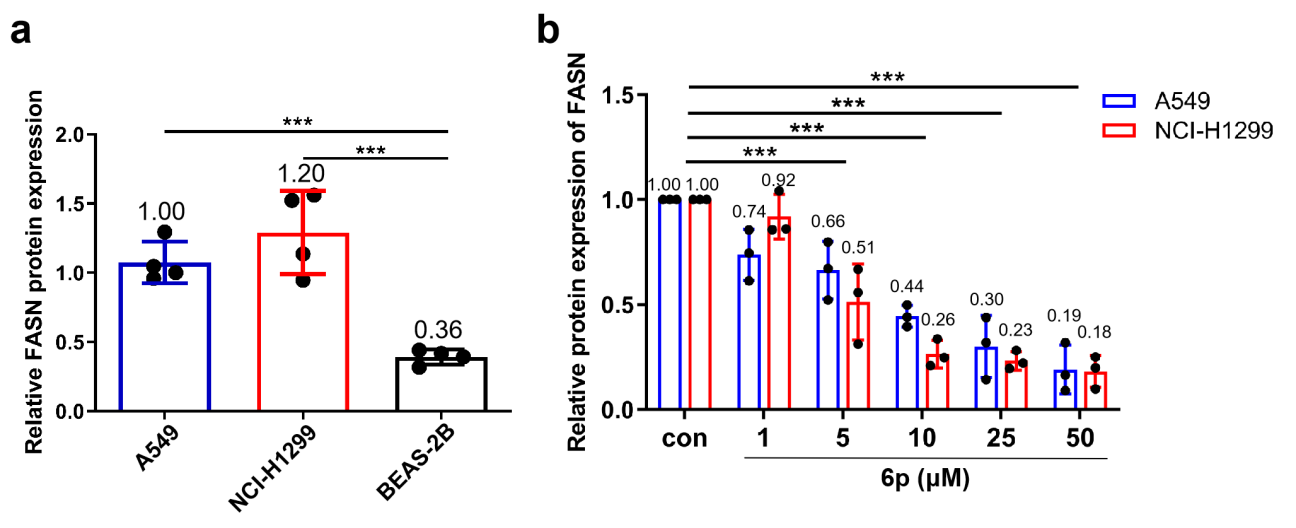


**Figure. S12.** **Compound 6p treatment decreases FASN expression in non-small cell lung cancer (NSCLC) cells. a** Relative FASN expression in NSCLC cell lines (A549 and NCI-H1299) and normal human bronchial epithelial BEAS-2B cell line (n = 4). **b** Relative FASN expression after treatment with 1, 5, 10, 25, and 50 μM **6p** for 48 h (n = 3). Data were shown as means ± SD, One-way ANOVA test. ****p* <0.001.

**Fig. S13.**


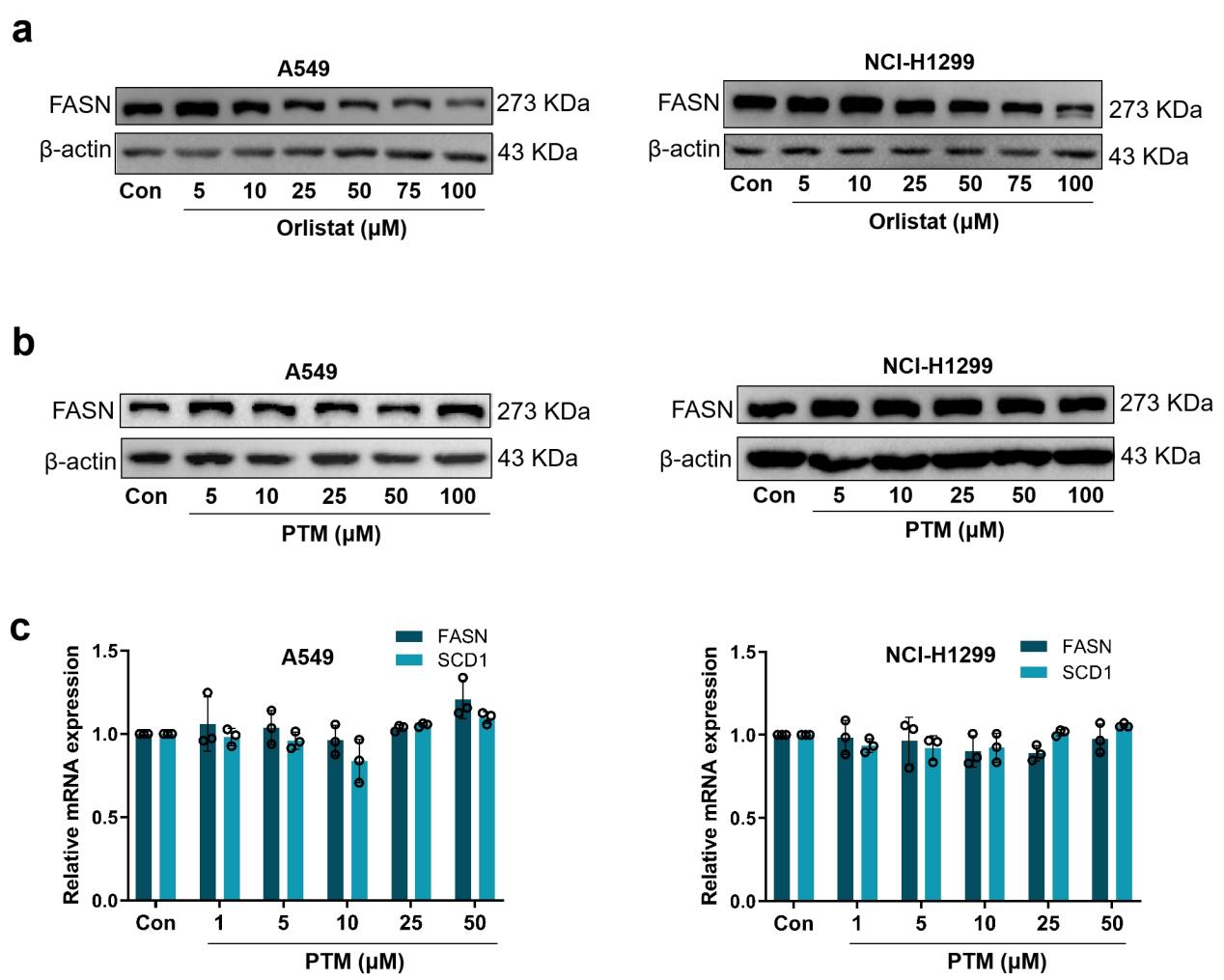


**Figure. S13. The FASN expression levels after orlistat and platensimycin (PTM) treatment. a** Western blot analysis of FASN protein expression after treatment with various concentrations of orlistat for 48 h. **b** Western blot analysis of FASN protein expression after treatment with various concentrations of platensimycin for 48 h. **c** RT-qPCR showed no changes in the mRNA expression of FASN and SCD1 when A549 and NCI-H1299 cells treated with platensimycin for 48 h (n = 3).

**Fig. S14.**


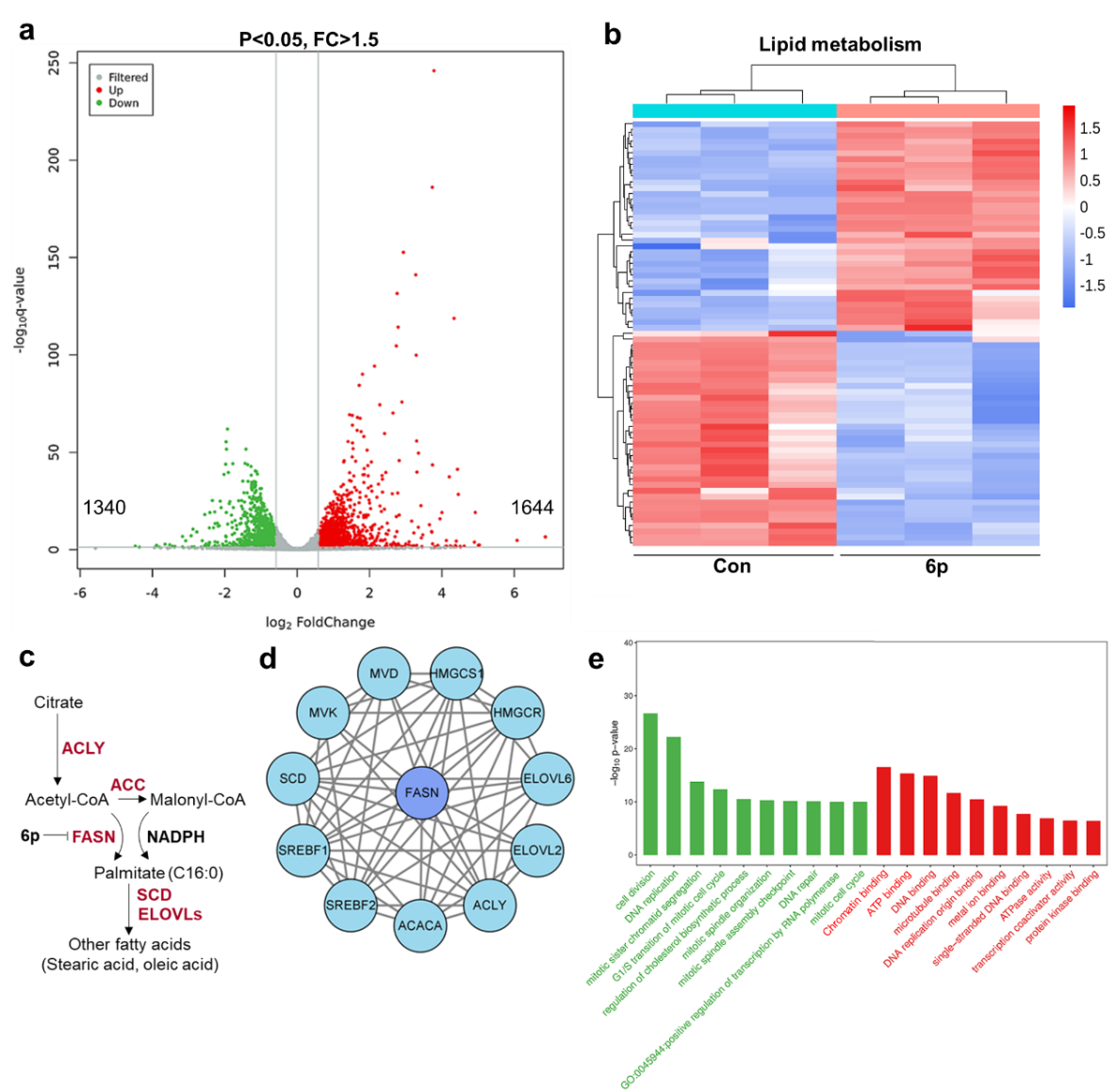


**Figure. S14. Compound 6p modulates lipid metabolic and growth-associated pathways in NSCLC cells. NCI-H1299 cells treated with 6p for 48 h were analyzed for gene expression using RNA sequencing. a** The genes with significant changes were identified using the statistical (*p* <0.05) and fold change (FC >1.5) criteria, including 1644 up-regulated genes and 1340 down-regulated genes. **b** Differentially expressed genes in lipid metabolism were identified between control and **6p**-treated cells. **c** Selected key enzymes in the *de novo* lipid synthesis pathway. **d** Protein-protein interaction networks of key enzymes in the *de novo* lipid synthesis and cholesterol synthesis pathway by STRING and Cytoscape software. **e** Functional annotation of significantly changed down-regulated genes by gene ontology analysis. Biological processes (green), molecular function (red). The bar graph showed the number of proteins in each gene ontology category, corresponding - log10 *p*-value.

**Fig. S15.**


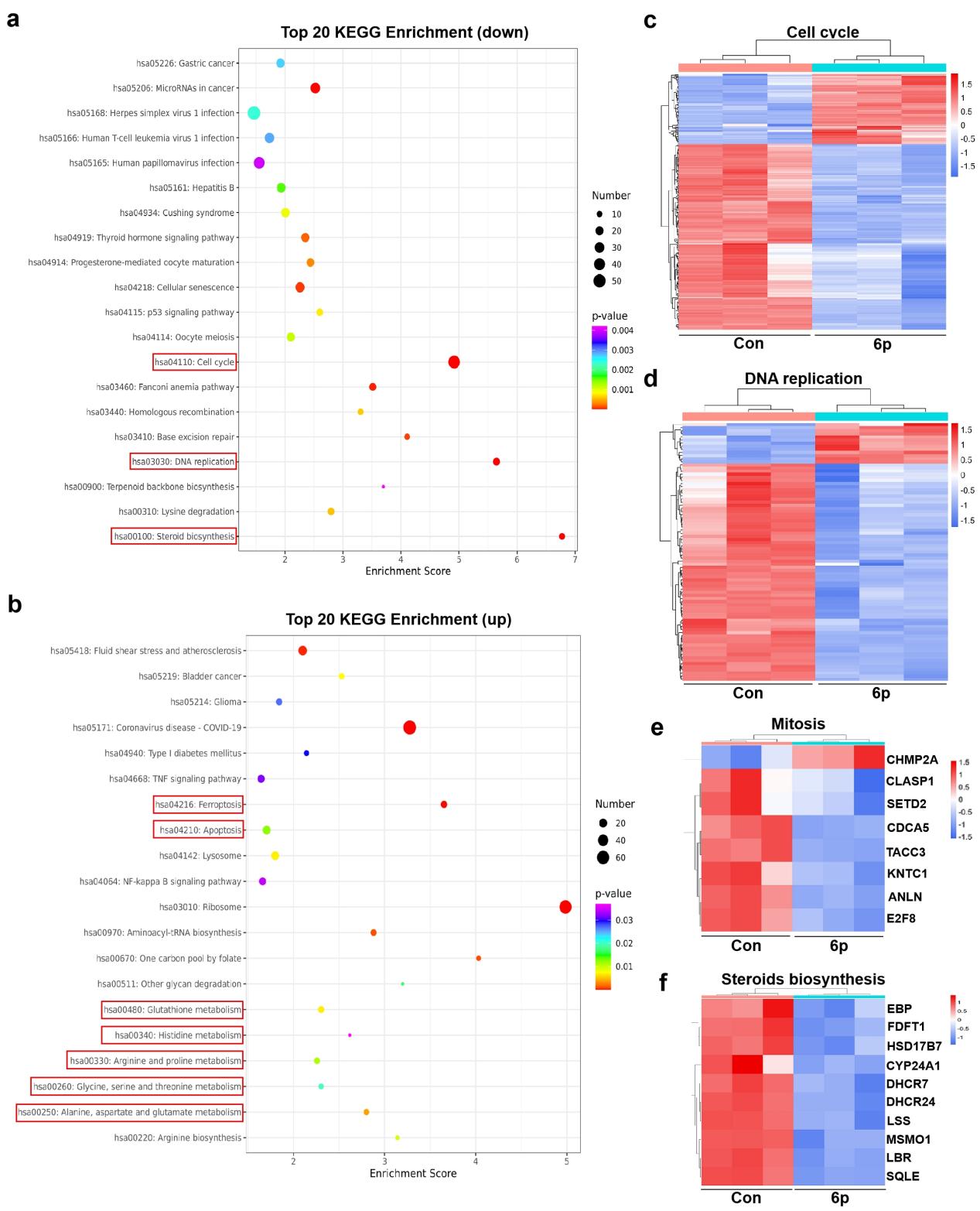


**Figure. S15. RNA-seq data analysis for NCI-H1299 cells treated with 6p. a** Down-regulated KEGG pathways of NCI-H1299 cells treated with **6p** of 25 μM for 48 h (Top 20). **b** Up-regulated KEGG pathways of NCI-H1299 cells treated with **6p** of 25 μM for 48 h (Top 20). **c** Differentially expressed genes in cell cycle. **d** Differentially expressed genes in DNA replication. **e** Differentially expressed genes in mitosis. **f** Differentially expressed genes in steroid biosynthesis.

**Fig. S16.**


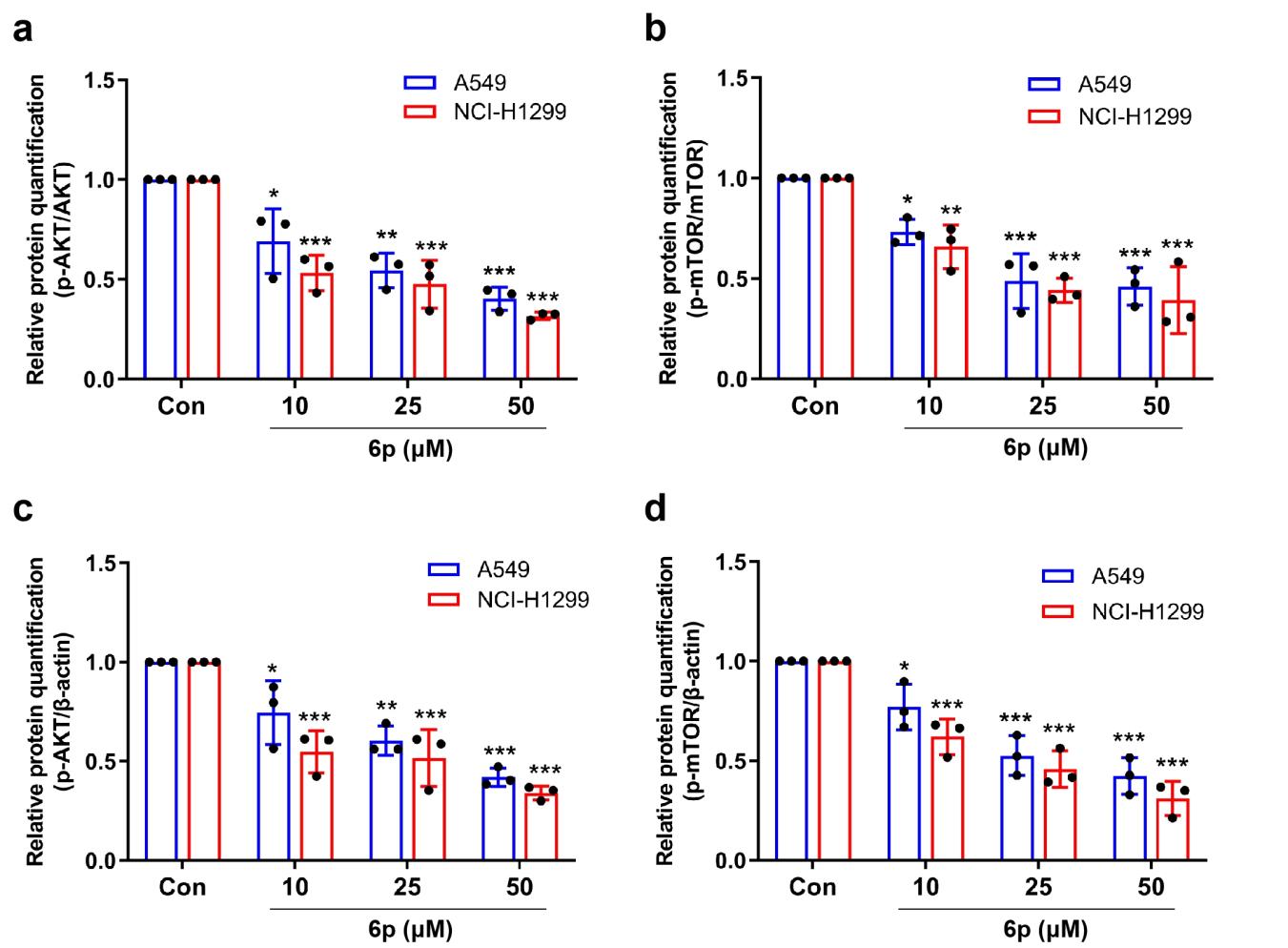


**Figure. S16. Compound 6p treatment decreases protein expression of PI3K-AKT-mTOR pathway in NSCLC cells. a** Western blot analysis of p-AKT/AKT protein expression with 10, 25, and 50 μM **6p** for 48 h (n = 3). **b** Western blot analysis of p-mTOR/mTOR protein expression with 10, 25, and 50 μM **6p** for 48 h (n = 3). **c** Western blot analysis of p-AKT/β-actin protein expression with 10, 25, and 50 μM **6p** for 48 h (n = 3). **d** Western blot analysis of p-mTOR/β-actin protein expression with 10, 25, and 50 μM **6p** for 48 h (n = 3). Data were shown as means ± SD, One-way ANOVA test. **p* <0.05 versus Con group, ***p* <0.01 versus Con group, ****p* <0.001 versus Con group.

**Fig. S17.**


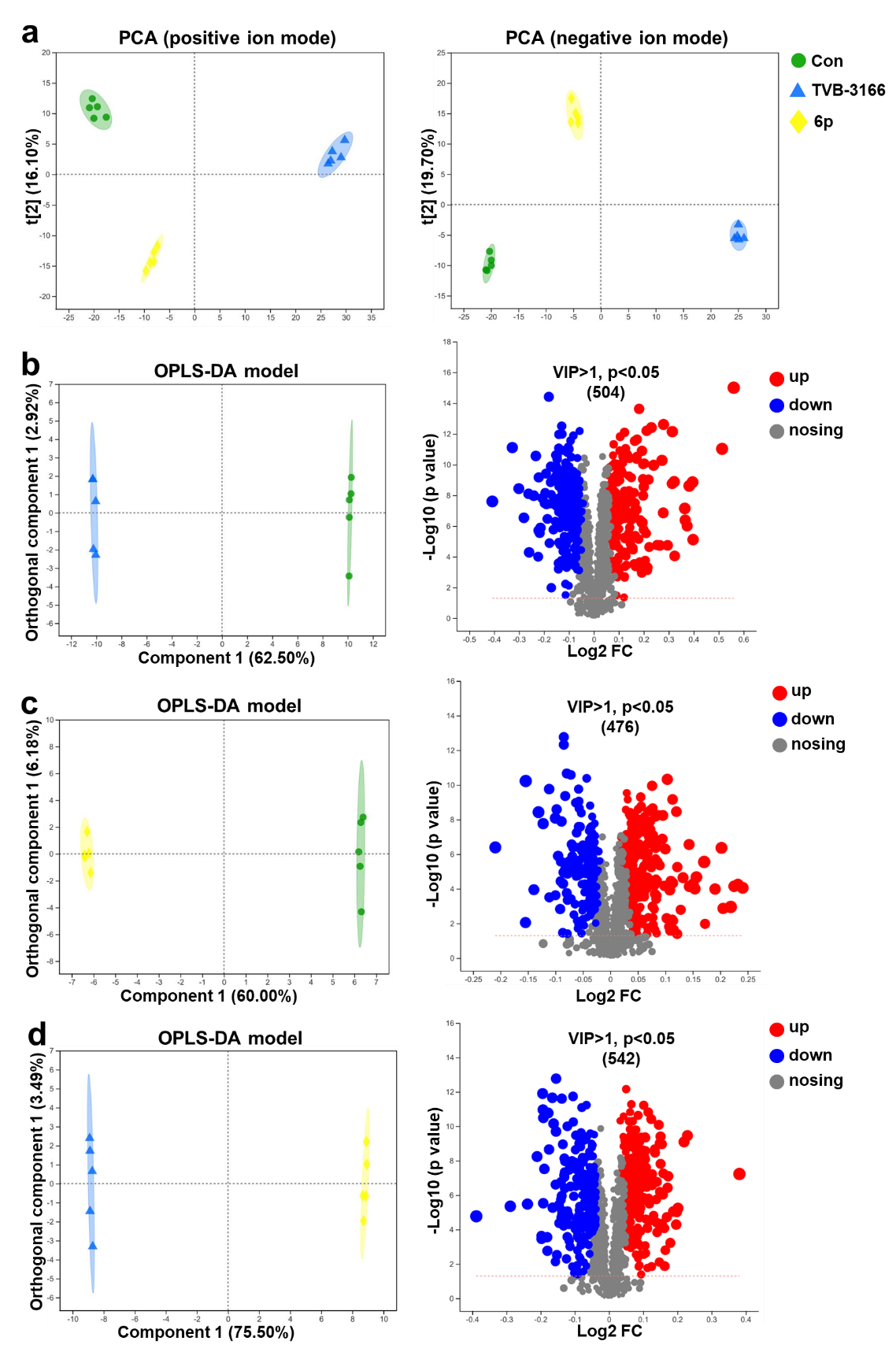


**Figure. S17. Multivariate data analysis for LC-MS lipidomics for 6p- and TVB3166-**

**treated NCI-H1299 cells. a** Unsupervised principal component analysis (PCA) scores plot. **b, c and d** Supervised OPLS-DA and differential lipid metabolites with VIP >1, *p* <0.05.

**Fig. S18.**

**
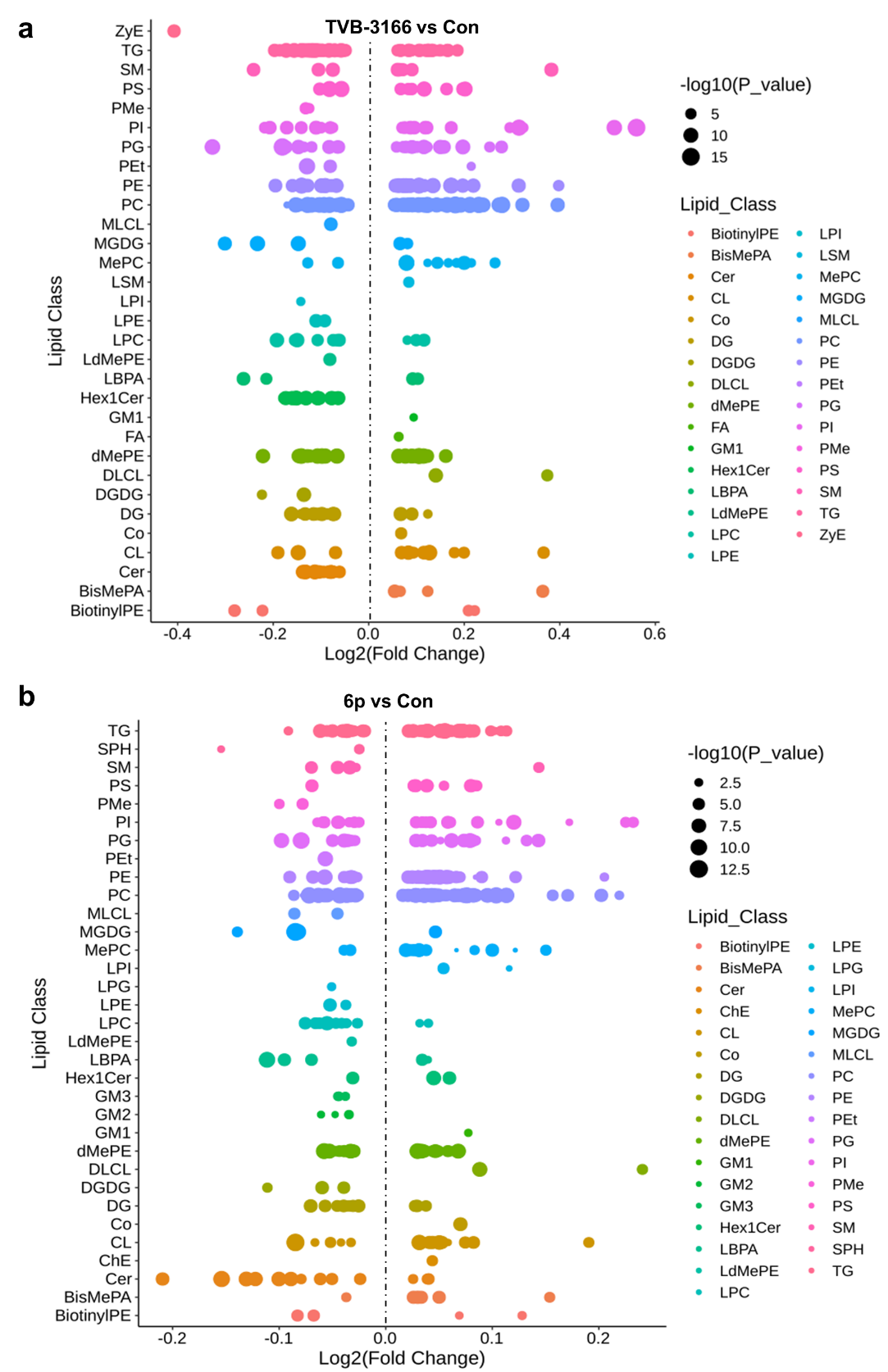
**

**Figure. S18. Log2 fold changes in lipid species. a** Log2 fold changes in lipid species in TVB-3166-treated versus control group. **b** Log2 fold changes in lipid species in **6p**-treated versus control group. The corresponding significance values displayed as -log10 (*p* value). Each dot represents a lipid species, and the dot size indicates significance. Only lipids with VIP >1, *p* <0.05 are displayed.

**Fig. S19.**


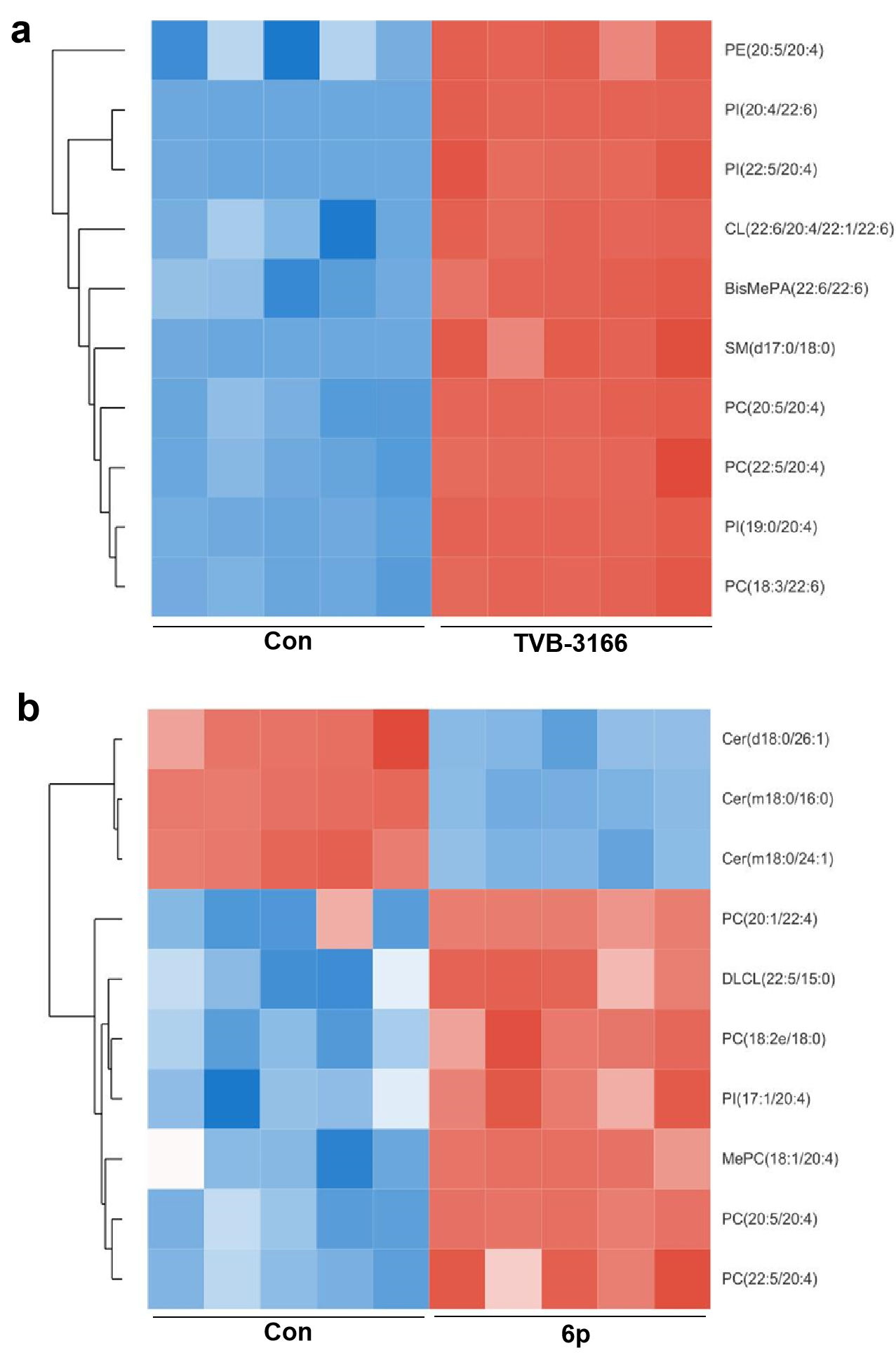


**Figure. S19. Compound 6p changes the abundance of lipid metabolites. a** The top 10 lipid metabolites according to the VIP value, detected in TVB-3166-treated and control groups. **b** The top 10 lipid metabolites according to the VIP value, detected in **6p**-treated and control groups.

**Fig. S20.**


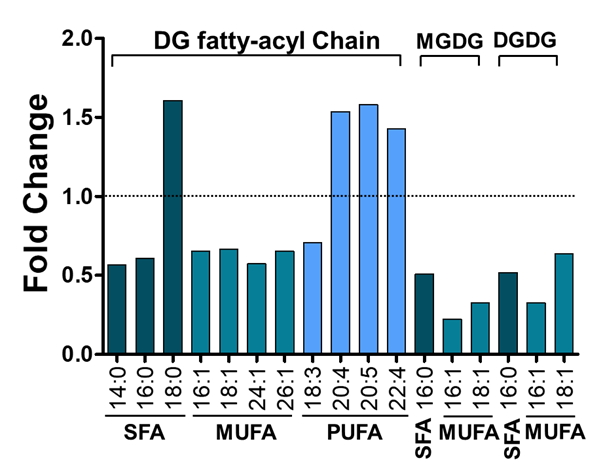


**Figure. S20. Compound 6p changes the fatty-acyl chains in other glycerolipids.** The intensity fold changes of individual fatty-acyl chains associated with diglyceride (DG), monogalactosyldiacylglycerol (MGDG) and digalactosyldiacylglycerol (DGDG) classes according to the VIP >1, *p* <0.05.

**Fig. S21.**


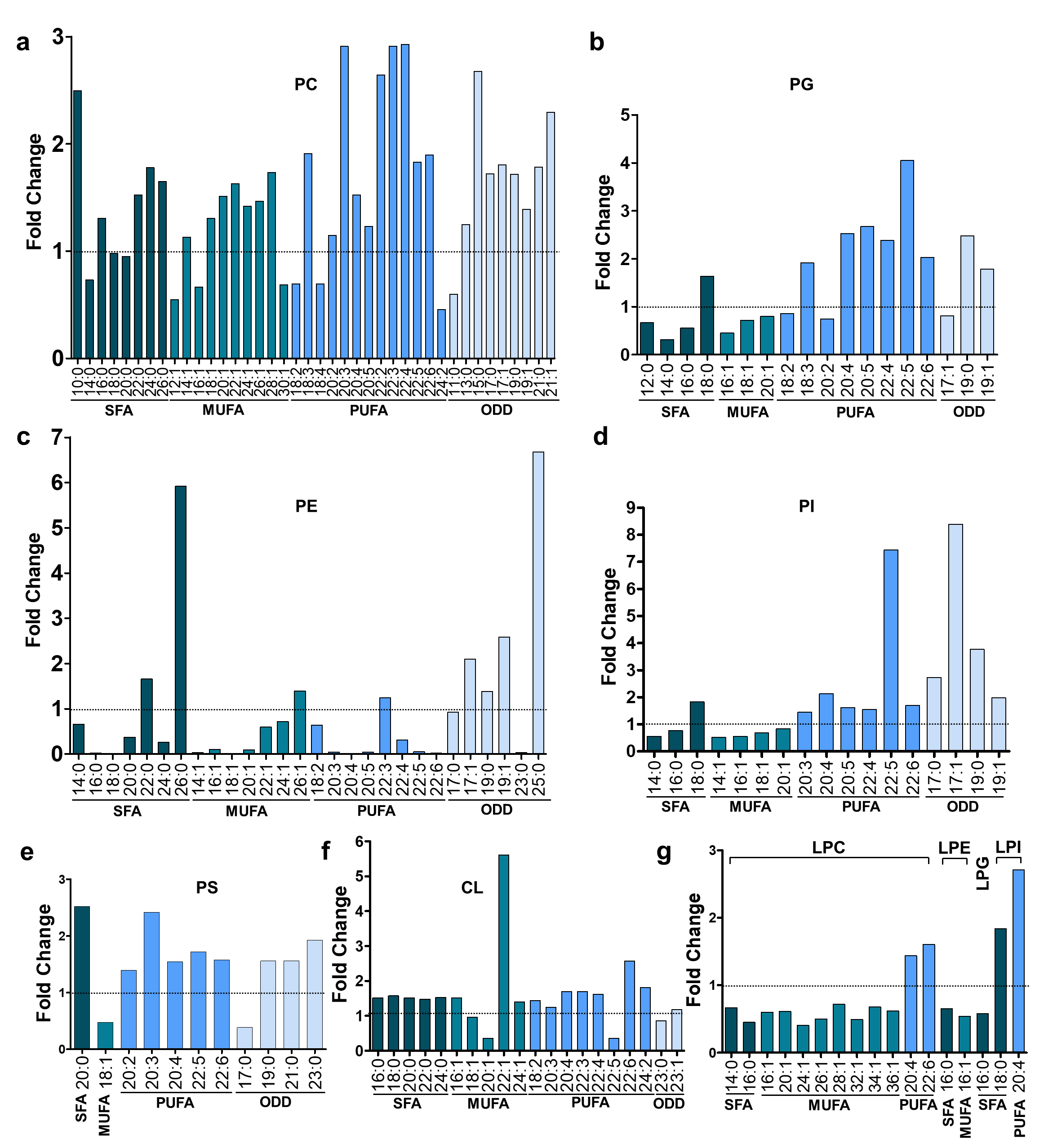
**Figure. S21. Compound 6p changes the fatty-acyl chains in** **glycerophospholipids. a-g** The intensity fold changes of individual fatty-acyl chains associated with different glycerophospholipid classes according to the VIP >1, *p* <0.05. SAF, saturated fatty acyls; MUFA, monounsaturated fatty acyls; PUFA, polyunsaturated fatty acyls containing two or three to six double bonds; ODD, odd-numbered fatty acyls.

**Fig. S22.**


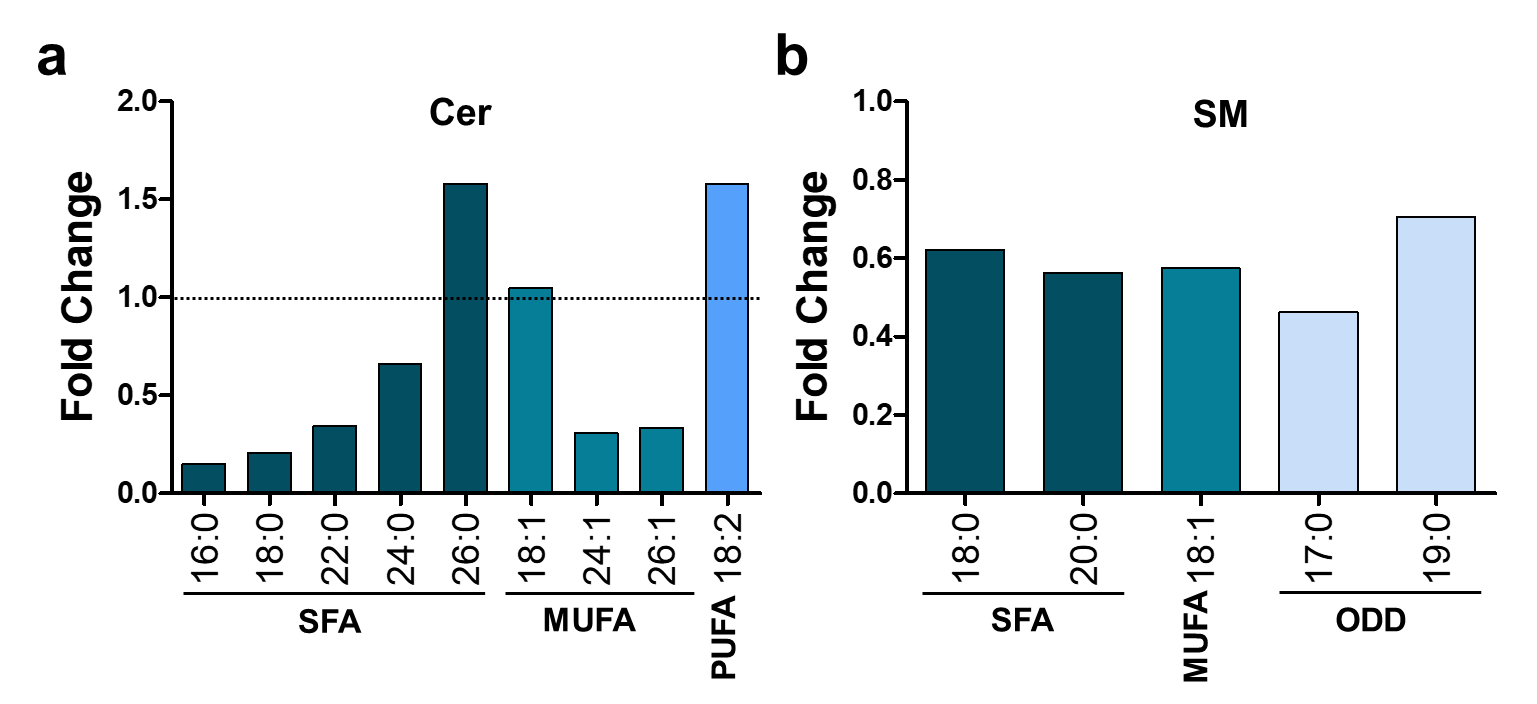


**Figure. S22. Compound 6p changes the fatty-acyl chains in sphingolipids. a-b** The intensity fold changes of individual fatty-acyl chains associated with different sphingolipid classes according to the VIP >1, *p* <0.05.

**Fig. S23.**


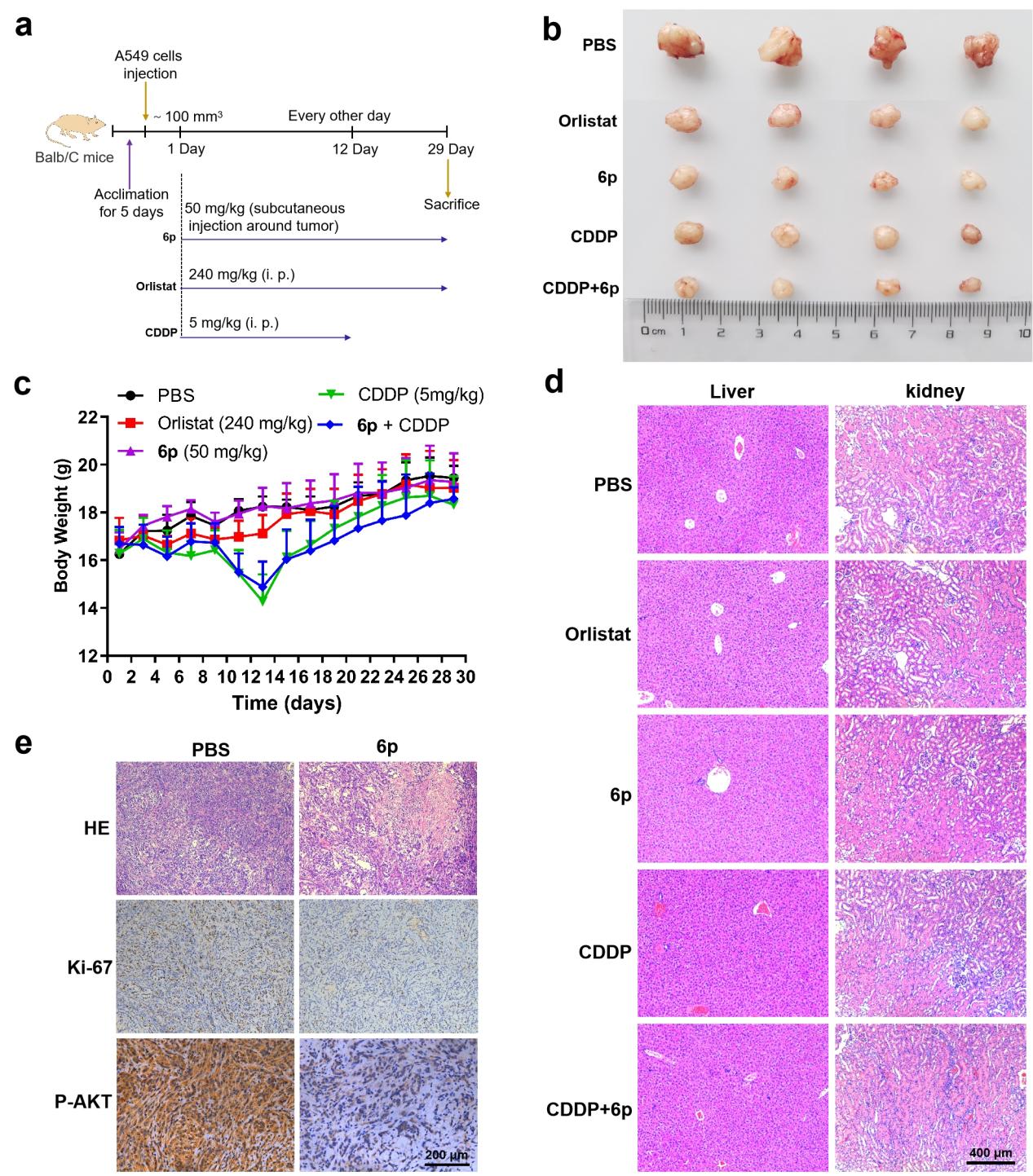


**Figure. S23. The in vivo therapeutic effect of 6p in A549-derived xenografts. a** The scheme for treatment assessment in the subcutaneous A549 tumor-bearing mice model. **b** Images of A549 xenografts receiving various therapies after sacrifice on day 29. **c** The mouse body weight during the treatment (n = 5). **d** HE staining of the liver and kidney tissues. **e** HE staining of the representative xenografts and immunohistochemistry assays involved in Ki-67 and p-AKT.

**Fig. S24.**


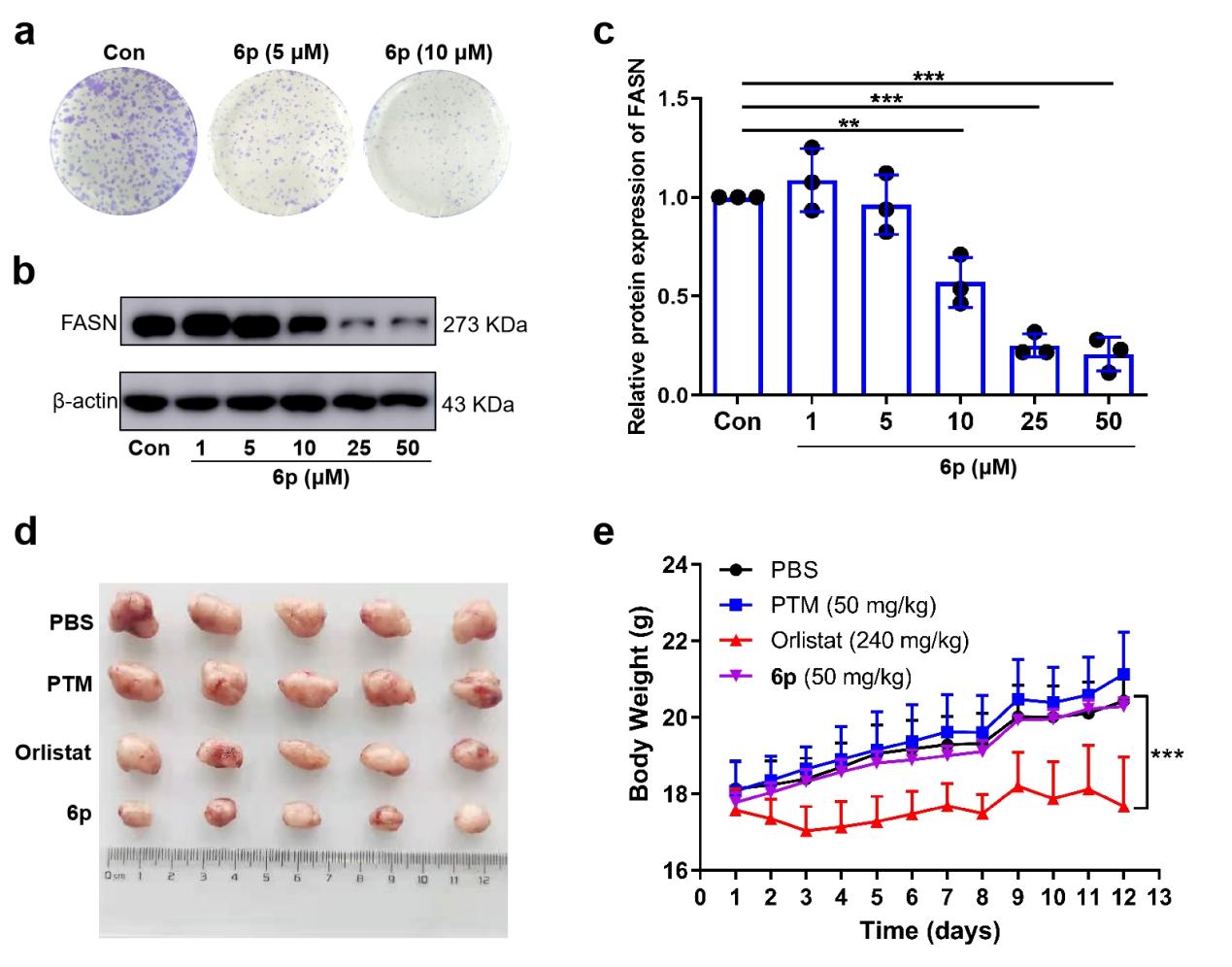


**Figure. S24. The in vitro and in vivo therapeutic effect of 6p in A375 cell lines and A375-derived xenografts, in comparison to orlistat and platensimycin (PTM). a** Inhibition of colony formation. **b and c** A375 cells were treated with various concentration of **6p** for 48h, western blot analysis of FASN protein expression (n = 3). **d** Images of A375 xenografts receiving various treatment after sacrifice on day 12. **e** The mouse body weight during the treatment (n = 5). Data were shown as means ± SD, One-way ANOVA (**c**) and Two-way ANOVA test (**e**), **p* <0.05, ****p* <0.001.

**Fig. S25.**


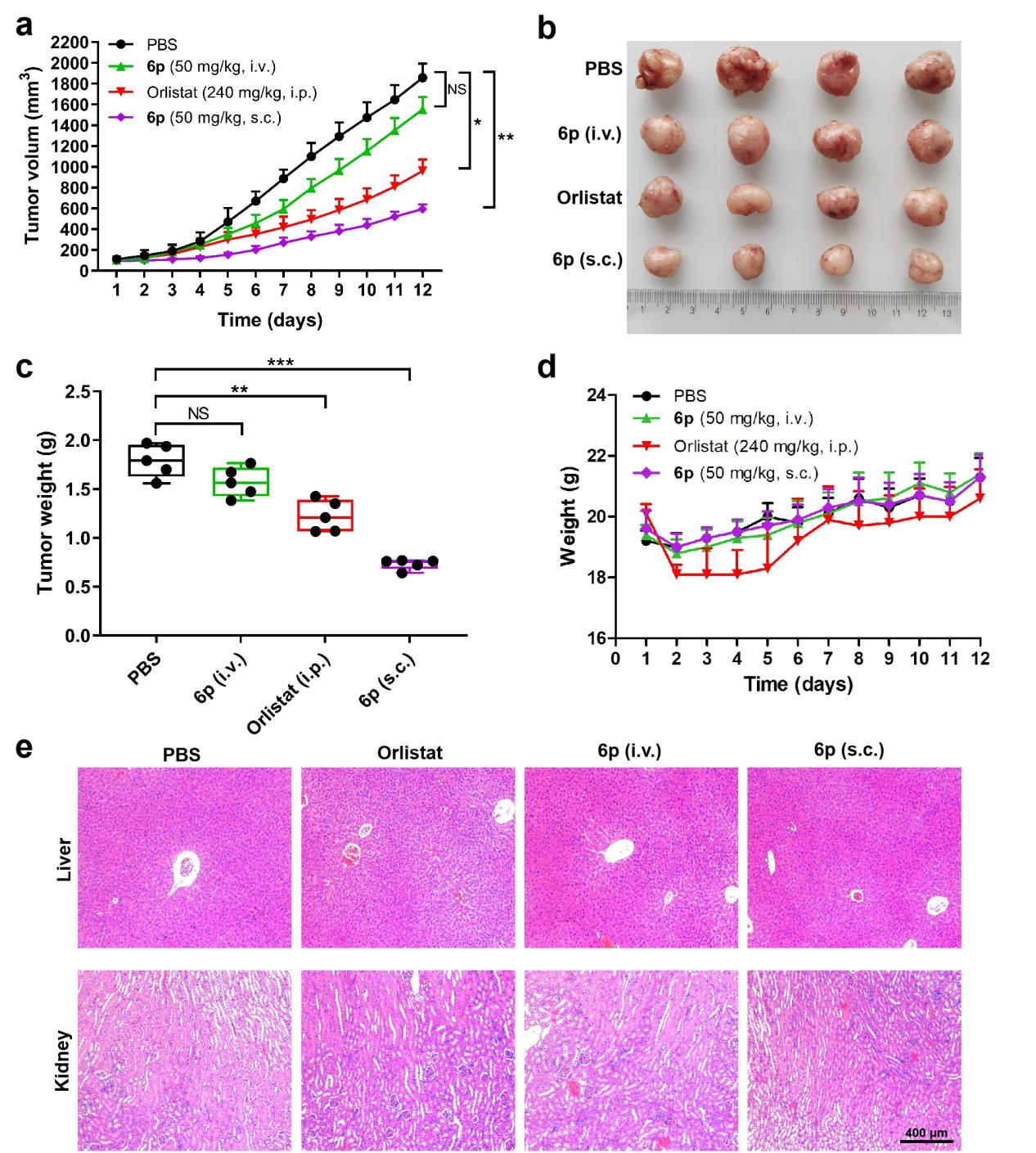


**Figure. S25. The comparison of therapeutic effects of 6p through intravenous administration and subcutaneous administration adjacent to the tumor in A375-derived xenografts. a** The tumor volume during the treatment (n = 5). **b** Images of A375 xenografts receiving various therapies after sacrifice on day 12. **c** Tumor weights (n = 5). **d** The mouse body weight during the treatment (n = 5). **e** HE staining of liver and kidney tissue sections. Data were shown as means ± SD, Two-way ANOVA test (**a**) and One-way ANOVA (**c**), **p* <0.05, ***p* <0.05, ****p* <0.001.

**References**

1. Jin, Z. J. About the evaluation of drug combination. *Acta Pharmacol Sin*. **25**, 146-147 (2004).

2. Xiong, Y. et al. Surfactin ameliorated the internalization and inhibitory performances of bleomycin family compounds in tumor cells. *Molecular pharmaceutics*. **17**, 2125-2134 (2020).

3. Ventura, R. et al. Inhibition of *de novo* palmitate synthesis by fatty acid synthase induces apoptosis in tumor cells by remodeling cell membranes, inhibiting signaling pathways, and reprogramming gene expression. *EBioMedicine*. **2**, 808-824 (2015).

4. Wang, H. C. et al. Therapeutic efficacy of FASN inhibition in preclinical models of HCC. *Hepatology*. **0**, 1-16 (2022).

5. Alwarawrah, Y. et al. Fasnall, a selective FASN Inhibitor, shows potent anti-tumor activity in the MMTV-Neu model of HER2 ^+^ breast cancer. *Cell Chemical Biology*. **23**, 1-11 (2016).

6. Zadra, G. et al. Inhibition of *de novo* lipogenesis targets androgen receptor signaling in castration-resistant prostate cancer. *PNAS*. **116**, 631-640 (2019).

7. You, B. J. et al. Orlistat displays antitumor activity and enhances the efficacy of paclitaxel in human hepatoma Hep3B cells. *Chemical Research in Toxicology*. **32**, 255-264 (2019).

8. Lee, K. H. et al. Inhibitory effect of emodin on fatty acid synthase, colon cancer proliferation and apoptosis. *Molecular Medicine Reports*. **15**, 2163-2173 (2017).

9. Deng, Y. C. et al. Morphing natural product platensimycin via Heck, Sonogashira, and One-Pot Sonogashira/cycloaddition reactions to produce antibiotics with in vivo activity. *Antibiotics*. **11**, 425 (2022).

10. Deng, Y. C. et al. Late-stage functionalization of platensimycin leading to multiple analogues with improved antibacterial activity in vitro and in vivo. *J Med Chem*. **62**, 6682-6693 (2019).

11. Qin, L. et al. Biomimetic stereoselective sulfa-Michael addition leads to platensimycin and platencin sulfur analogues against methicillin-resistant *Staphylococcus aureus*. *J. Nat. Prod*. **81**, 316-322 (2018).

12. Qiu, L. et al. A facile semi-synthetic approach towards halogen-substituted aminobenzoic acid analogues of platensimycin. *Tetrahedron*. **73**, 771-775 (2017).

13. Deng, Y. C. et al. The semi-synthesis, biological evaluation and docking analysis of the oxime, hydrazine and hydrazide derivatives of platensimycin. *Med. Chem. Comm*. **9**, 789-794 (2018).

14. Deng, Y. C. et al. Semisynthesis of platensimycin derivatives with antibiotic activities in mice via Suzuki−Miyaura cross-coupling reactions. *J Med Chem.* **61**, 11341-11348 (2018).
